# Modeling Gas Adsorption and Mechanistic Insights into Flexibility in Isoreticular Metal–Organic Frameworks Using High-Dimensional Neural Network Potentials

**DOI:** 10.1021/acs.langmuir.4c04578

**Published:** 2025-03-14

**Authors:** Omer Tayfuroglu, Abdulkadir Kocak, Yunus Zorlu

**Affiliations:** Department of Chemistry, Gebze Technical University, 41400 Gebze, Kocaeli, Turkey

## Abstract

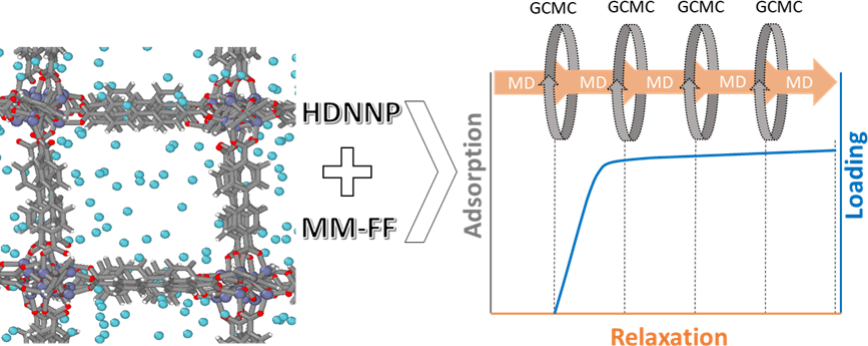

Metal–organic frameworks (MOFs), known for their remarkable porous and well-organized structures, have found extensive use in various applications, including gas storage. Predicting the bulk properties from atomistic simulations as well as gas uptakes and the adsorption mechanism requires the most accurate definition of MOF systems. The application of ab initio molecular dynamics to these extensive periodic systems exceeds the current computational capabilities. Consequently, alternative strategies need to be devised to decrease computational costs without compromising accuracy. In this work, we construct high-dimensional neural network potentials (HDNNPs) to describe rotationally and translationally invariant energies and forces of isoreticular metal–organic framework (IRMOF) series at the density functional theory level of accuracy using a fragmentation technique to study H_2_ and CH_4_ adsorption isotherms by means of an “adsorption–relaxation” model in which molecular dynamics and grand canonical Monte Carlo simulations were performed simultaneously. Herein, for the first time, we report that HDNNPs could be utilized for such simulations with excellent agreement with experimental values. We also report that the UFF4MOF force field may not be suitable for adsorption–relaxation simulations. In addition, we show that the real number of CH_4_ uptake values of IRMOF-10 under the extreme conditions could be much greater than what the classical force field predicts. Adsorption–relaxation simulations enable us to characterize the behavior of MOF atoms and the distribution of gas molecules during the adsorption process, giving the most detailed mechanistic picture.

## Introduction

Metal–organic frameworks (MOFs) have attracted a great deal of scientific research focus due to their variety of applications ranging from gas storage^1−5^ and separation^6−9^ to drug delivery,^10−13^ catalysis,^14,15^ and sensors.^16^ These and many more diverse applications are possible mainly because of the MOFs’ highly tunable physical and chemical properties arising from their vast number of plausible structures by the combination of a variety of available inorganic and organic building blocks. One of the aspects of MOFs is the utilization of their flexibility, distinguishing them from rigid porous materials like zeolites,^17^ for performance improvements in many applications such as gas storage and separations. The flexibility, which can be extrinsic or intrinsic,^18^ causes reversible arrangements of organic and inorganic subunits such as expanding, contracting,^19^ gate opening, and breathing effects^20^ under mechanical/thermal stress or host–guest interaction.^21^

On one hand, experimental characterization of materials must be conducted and will always be vital in understanding the true physical and chemical properties of materials. On the other hand, quite large numbers of MOFs require too much experimental effort and cost for the characterization of such materials. In addition, the atomistic details of the host–guest interaction may not even be possible with existing experimental techniques. In this regard, this problem can be sorted out with the help of computational studies. Atomistic simulations not only provide complementary information but also explain experiments.

Computational studies on the gas adsorption properties of MOFs mostly rely on classical force fields. Classical force fields suffer from the coarse definition (molecular mechanics (MM) and so forth) of MOFs. In addition, the classical force fields use predefined parameters, and these parameters do not change throughout the simulation. Indeed, there is an active research area for developing classical force fields to consider the flexibility of MOF systems.^22^ The QuickFF,^23,24^ MOF-FF,^25,26^ BTW-FF,^27^ ZIF-FF,^28^ and UFF4MOF^26^ are some examples of classical force fields, with the first two being designed for specific MOFs and the rest being generalized to all MOFs. All of these methods still lack electron-involved phenomena such as bond formation or breaking, and the bonded potentials are limited to classical harmonic behavior. On the other hand, density functinal theory (DFT) methods with inclusion of periodic boundary conditions are much better for mimicking bulk properties of MOFs since short-range local environments of atoms are defined more accurately. There have been numerous DFT studies on MOFs with various aspects such as electronic and vibrational properties,^29,30^ catalytic behavior,^31^ and gas adsorption profiles.^32,33^ However, first-principles definition of MOFs, which includes thousands of atoms in unit cells, is still a challenging task in terms of computational cost. Long simulation times are required since extensive sampling of the system is a necessity for accounting for the dynamic definition of physical and chemical processes such as adsorption, desorption, and diffusion of gas molecules, which altogether make ab initio simulations prohibitively expensive. A trade-off between the accuracy and cost is to use machine learning (ML) potentials that are trained by DFT methods.

Over the past decade, machine learning (ML) has emerged in a variety of disciplines to cover the gap between the most accurate ab initio and fast classical simulations.^34−39^ MOFs can be described by the construction of highly accurate and reactive ML potentials to represent multidimensional potential energy surfaces (PESs) to perform much more accurate simulations with enormous computational acceleration.^34,40−45^

To date, several ML potentials have been constructed using different artificial neural network (ANN) architectures based on the atomic contribution to the desired property. ANI-torch,^46^ high-dimensional neural network (HDNNP),^47,48^ deep tensor neural network (DTNN),^36^ and SchNet^34^ are the most common ANNs that have been used to train PESs. Pioneering work on the construction of neural network potentials (NNPs) with an HDNN architecture for isoreticular metal–organic framework-1, IRMOF-1 (MOF-5), was reported by Behler and co-workers.^36^ Recently, we have reported the construction and application of a generic NNP for the IRMOF-*n* series (*n* = 1, 4, 6, 7, or 10) trained by PBE-D4/def2-TZVP reference data of MOF fragments.^49^ The “fragmentation technique” is employed to train bulk MOFs by decomposing them into representative fragments. This approach allows for the inclusion of diverse atomic environments, with each fragment capturing the local chemical surroundings within the bulk structure. HDNNPs trained on these fragment environments are then able to approximate the properties of the entire bulk system, including its total energy. For a detailed description of the fragmentation process and NNP construction, see the relevant literature.^36,49^ In addition, Johnson and co-workers^22^ developed an ML potential for UiO-66 and applied these potentials for modeling gas diffusion behaviors via molecular dynamics simulations.

Current methods for studying gas uptake of porous materials rely on the grand canonical Monte Carlo (GCMC) simulations since they allow both the translational/rotational motions and insertion/deletion of the guest molecules. However, the flexibility of MOFs limits the GCMC simulations since the adsorbent atoms are usually fixed during the GCMC steps (“rigid adsorption”) due to enormous cost.^50^ To extensively sample the flexibility of MOFs, MD simulations could be performed instead. Nonetheless, MD does not allow the number of adsorbed particles to change during MD steps.^51−53^ Therefore, a single MD simulation is not sufficient in studying adsorption isotherms. Thus, for modeling the dynamic behavior of MOFs to sample the flexibility of the host along with the adsorption of guest molecules, MD simulations can be incorporated with GCMC simulations. Combination of MD and GCMC simulations not only reflects the extrinsic flexibility, triggered by external stimuli such as mechanical stress and gas adsorption that causes swelling, breathing, etc., but also allows the observation of intrinsic flexibility, due to thermal vibrations even in the absence of external stimuli during gas adsorption on MOFs.^18^ A few attempts have been made to effectively select the simulation protocol in which classical FF-based GCMC and MD are combined.^54,55^ Using this combination, Ghofi and Maurin have studied phase transitions of MIL-53 as an extrinsic flexibility for CO_2_ adsorption.^56^

**Figure 1:**
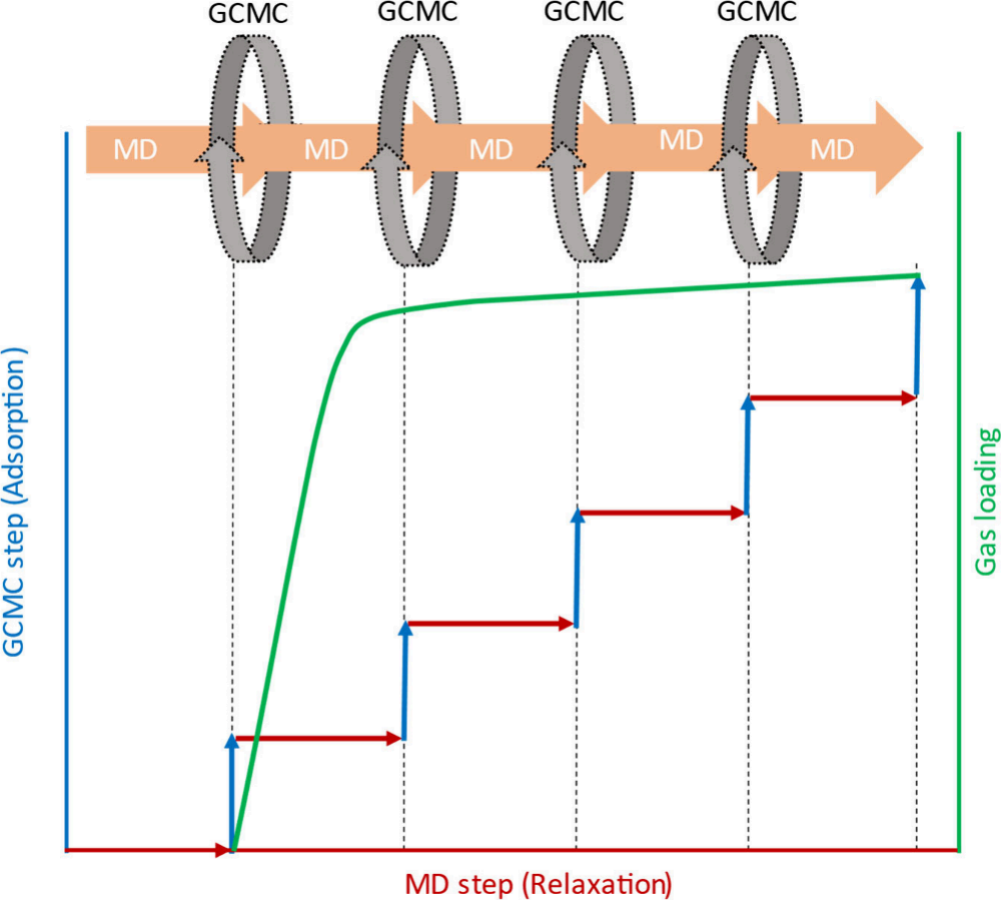
Depiction of adsorption–relaxation simulations. GCMC insertion/deletion of guest molecules occurs on the fly during NPT–MD simulations. Gray circles and pink arrows (top) indicate GCMC and MD simulations, respectively. Red, blue, and green colors (bottom) represent adsorption during MC steps, relaxation during MD steps, and gas loadings, respectively.

To the best of our knowledge, currently there are two specific approaches^57^ to combine MD simulations with GCMC simulations: “flexible snapshot adsorption” and “adsorption–relaxation”. In the first approach, snapshots are taken from the trajectory of the MD simulations of a bare MOF (without guest molecules), and GCMC simulations are performed for gas adsorption for each snapshot. In the second approach, GCMC gas insertion and deletion occur on the fly during the MD simulation as depicted in Figure 1. Accounting for the flexibility of MOFs is best represented by the adsorption–relaxation model.^57^ Although the flexible snapshot adsorption model somewhat reflects the flexibility and benefit in relation to computational cost, this method is not realistic, since it does not allow dynamic interaction of adsorbates with the framework atoms. Gas adsorption is calculated at bare MOF snapshots instead of allowing the MOF to respond to the guest molecules as they traverse along the window.

Recently, Sholl and co-workers have reported the screening of MOFs for gas uptake using combined MD/GCMC simulations using the adsorption–relaxation model, which accounts for both extrinsic and intrinsic flexibility (i.e., adsorption-induced flexibility).^57^ However, they have used classical force fields, which limited their simulations to classical harmonic vibrations in accounting for the intrinsic flexibility. Among their studied MOFs, MIL-53, which has a phase transition to possess either large pores or small pores, was reproduced very well with MD/GCMC simulations, whereas rigid defined MIL-53 in either form was too off from the experimental results. On the other hand, IRMOF-1, which has rather low extrinsic flexibility, behaved almost identically to the rigid definition. In addition, MD/GCMC on UiO-66 did not show a significant difference from calculations assuming a rigid framework. This might be due to the limitation of the classical force field used in these simulations, as the studies have suggested UiO-66 exhibits considerable flexibility. The latter has also been studied by Johnson and co-workers^22^ using an NNP as the force field of the framework in the MD simulations by means of diffusion of adsorbates. This showed the superiority of using an NNP with near-DFT accuracy over classical force fields in describing MOF structures. They also showed the feasibility of using the hybrid potential as an NNP combination with classical force fields in simulations. They successfully applied an NNP for interatomic interactions of a bare MOF while using a classical force field for adsorbate–adsorbate interactions along with combining rules to account for adsorbent–adsorbate interactions. However, to the best of our knowledge, no report in the literature has used NNPs when defining all or part of the system during the adsorption–relaxation simulations (i.e., interactive coupling of GCMC with MD simulations).

The best option for performing these adsorption–relaxation simulations is to define all of the systems with NNPs. Although DFT calculations can be performed in the presence of guest molecules in MOFs to train MOF–adsorbate and adsorbate–adsorbate interactions, this can be computationally too expensive, as it requires a massive training set that includes different gases and their mixtures or a new NNP for each MOF–gas pair. Due to this challenge, almost none of the NNPs reported in the literature account for the MOF–adsorbate and adsorbate–adsorbate interactions that allow direct use of NNPs to study the adsorption or diffusion properties of gases on MOFs. An alternative strategy to this challenge is to use NNP/MM hybrid potentials in which adsorbate–adsorbate interactions, which consist of mostly van der Waals interactions, can be defined using classical force fields, which is known to perform very well in simple fluids and mixtures while nonbonded framework–adsorbate interactions (atom–atom or atom–united atom) are defined by combining rules.^22,58^ Thus, hybrid potentials such as an NNP for a MOF and a classical FF for an adsorbate could be incorporated into adsorption–relaxation simulations.

In this study, we investigate methane and hydrogen gas adsorption profiles on IRMOF-1 and IRMOF-10 in the most extensive mechanistic detail, for the first time, utilizing HDNNP in the definition of the MOF structure in combined GCMC/MD (i.e., adsorption–relaxation) simulations. These simulations allow the most realistic and accurate (at DFT accuracy) behaviors of MOFs during the on-the-fly gas adsorption process. Our findings also suggest that the most used UFF4MOF definition for MOF structures may not be suitable for adsorption–relaxation simulations due to the distortion of the MOF structure induced by adsorbates.

## Computational Methods

### HDNNP Construction

We have reconstructed the HDNNP by n2p2 library using the original data set (from our previous report^59^), suitable for the LAMMPS simulation package. To address cost and memory constraints, we utilized a reduced version (∼98K structures) of the original data set (∼173K structures) to train the HDNNP. Redundancy in the data was minimized by employing K-means clustering based on the root-mean-square deviation (RMSD) of the three-dimensional geometries, allowing us to select representative structures from each cluster. This approach ensured efficient training with minimal data loss, as the n2p2 framework maintains accuracy with fewer data points and higher memory efficiency compared to that of SchNet.

A LAMMPS suitable n2p2 C++ library^60,61^ was used with the neural network potential formalism of Behler and Parrinello (BPNN)^62^ to train interatomic potentials. BPNN uses symmetry functions to describe atomic environments, and the total energies and forces are calculated by the summation of pairwise interatomic interactions. We generated symmetry functions with a cutoff radius for atomic environments of 6.0 Å. For the training, multistream Kalman filter parameters were used as implemented in the n2p2 library as recommended by Singraber et al.^60^ The only exception to these parameters was that we used a fixed ratio of energy to force updates of 1:15.

### Modeling the System by NNP/Classical FF

After constructing the HDNNP, we readily studied combined MD/GCMC simulations at different pressures (in the range of 1–100 bar at regular intervals) for loading H_2_ and CH_4_ gases on each IRMOF-1 and IRMOF-10 via the adsorption–relaxation model. Here, MOFs were modeled by our NNP, whereas remaining interactions such as gas–gas and gas–MOF interactions were modeled by classical FF definitions.^26^ The adsorbent gas molecules were assumed to be spherical Lennard-Jones fluids with no charges. The adsorbent–adsorbent interactions were modeled with the LJ potential.
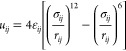
where *u*_*ij*_ is the potential energy, *ε*_*ij*_ is the well depth, *σ*_*ij*_ is the cutoff diameter, and *r*_*ij*_ is the distance between two spherical gas molecules. Intramolecular interactions of the MOFs are defined using our NNP, while gas–gas and gas–MOF interactions are modeled using parameters from ref (63). Specifically, the Lennard-Jones parameters for CH_4_ are taken from TraPPE-UA,^64^ and those for H_2_ are derived from ref (65). For gas–MOF interactions, the Lennard-Jones parameters for the MOFs are sourced from UFF,^66^ with Lorentz–Berthelot combining rules applied to these interactions ( and . The cutoff distance for all LJ interactions was set to 12 Å, with tail energy corrections applied. The LJ parameters for gases and MOFs are given in Table S1.

In the case of classical FF simulations, the dynamic properties of MOFs were modeled by UFF4MOF, whose accuracy has been demonstrated by comparing it to ab initio-derived force fields by Moghadam et al.^26^ Since UFF4MOF does not include partial charges on MOF atoms, the preassigned atomic charges of MOFs were retrieved from ref (67). The nonbonding interactions among MOF atoms were defined as the Coulombic and Lennard-Jones terms, whereas MOF–gas and gas–gas interactions use only LJ potentials. For the LJ interactions, the same parameters (i.e., 12 Å cutoff and Lorentz–Berthelot combining rules) were used.

### GCMC/MD Protocol

For the adsorption–relaxation flexible adsorption simulations on MOFs, we used a GCMC-altered MD simulation (GCMC/MD) protocol in which GCMC and MD are performed in LAMMPS.^68,69^ Prior to gas adsorption simulations, we relaxed bare MOF structures by means of energy minimization using a conjugate gradient (CG) algorithm with energy and force thresholds of 10^–8^ eV and 10^–4^ eV/A, respectively. Following that, an *NVT* equilibration for 150 ps was performed at different temperatures of interest using the Nose–Hoover thermostat (i.e., 77, 200, and 300 K). Final structures were used in NPT–MD simulations, which are combined with GCMC simulations at 77 K for H_2_ and 300 K for CH_4_. In the NPT simulations, the Nosé–Hoover thermostat and barostat were defined with 0.5 fs time steps. Damping constants were set to 0.1 ps for both the thermostat and the barostat. One step of the GCMC simulation is performed in every 250 fs long MD simulation. GCMC cycles during the MD simulations were also performed in LAMMPS. Given that LAMMPS is primarily designed for MD simulations rather than GCMC, its performance was validated against the results from RASPA (Figure S1). During the GCMC step, 100 random exchanges of the adsorbate are attempted. Monte Carlo trial attempts were made by allowing translations, insertions, and deletions, all with equal probabilities. The rotation moves were turned off since the adsorbent molecules were assumed to be spherical. This GCMC alteration process is repeated until a total of 2 ns of MD simulation time (a total of 8000 GCMC steps altered during a 2 ns MD).

### GCMC Simulations for Rigid Definition

For the stand-alone GCMC simulations (not combined with MD), the RASPA software package^70^ was used. For these types of calculations, we refer to the MOFs as “rigid” throughout the manuscript.

In these calculations, structures in a 2 × 2 × 2 supercell were run for 1000 cycles with the first 500 being equilibration and the last 500 for the ensemble average. To model guest molecules, the single-site spherical Lennard-Jones (LJ) 12–6 potential taken from the TraPPE^64^ force field was used in the GCMC simulations. The positions of MOF structures were kept fixed during GCMC simulations with the CrystalGenerator force field (CG)^70^ mixing with the Lorentz–Berthelot combining rules to assess the LJ cross-term guest/host parameters. Periodic boundary conditions (PBCs) in all three directions were applied, and a 12 Å interaction cutoff distance was used. The methane adsorption calculations were conducted at 300 K and 100 bar, while for hydrogen, we used 77 K and 100 bar.

## Results and Discussion

### Validation/Test of HDNNP

After constructing the NNP, we assessed its success on both training and test data sets for fragments. A perfect agreement of HDNNP with DFT calculations was observed (Figures S2–S7). In addition, to evaluate the stability of HDNNP, 1 ns NVT–MD simulations following energy minimization at 300 K on periodic bulk IRMOF-1 and IRMOF-10 structures were run. During the MD simulations, all configurations visited were located within the extensively sampled regions of HDNNP, and no significant alterations in structure were observed, except for minor thermal fluctuations. The total energy of the system is conserved throughout the simulation (Figure S8). These simulations are performed in the absence of adsorbate gases and believably reflect the extrinsic flexibility but not adsorbate-induced flexibility, although both MOFs are quite rigid.

After ensuring our HDNNP’s success on fragments and smooth MD simulations, we also calculated equilibrium bulk properties as a final test of our HDNNP as a comparison to experimental and DFT-calculated values. To estimate bulk modulus and equilibrium lattice constants, we performed NPT–MD simulations for 2 ns using a Nose–Hoover barostat (at 1 bar). Prior to the final simulations, we first conducted energy minimization, followed by an NVT–MD simulation for 75 ps. The simulations were run at 100 and 300 K using a Nose–Hoover thermostat. The last 1 ns of the final simulations was used in the bulk property analysis. Table 1 shows the comparison of equilibrium lattice constants produced by HDNNP of bulk IRMOF structures with experimental single-crystal X-ray diffraction (SCXRD) values at 100 K^71^ along with our previously reported values.^59^ The HDNNP-calculated equilibrium lattice constants and bulk modulus values are in excellent agreement with experiments and our previously reported values, as well as literature calculations. The difference equilibrium lattice constants between experimental and HDNNP estimated values are only in the range of 0.14 and 0.41 Å for IRMOF-1 and IRMOF-10, respectively. Our HDNNPs have been trained from non-equilibrium geometries of fragments but can successfully predict the periodic equilibrium parameters. In addition to bulk properties, we have also calculated phonon modes and NTE properties to validate the performance of our HDNNP (Figures S9 and S10). Both phonons and NTE behavior are in perfect agreement with our previously reported values^49^ and experiments.

**Table 1:** Equilibrium Lattice Constants and Bulk Moduli of IRMOFs Calculated Using HDNNP and Compared with Those from Experimental SCXRD and Other Calculations in the Literature

	equilibrium lattice constant (Å)				bulk modulus (GPa)		
	SCXR (100 K)	HDNNP (100 K)	HDNNP (300 K)	simulations	HDNNP (100 K)	HDNNP (300 K)	simulations
IRMOF-1	25.89	26.03	25.95	26.05^a^ (0 K), 26.08^a^ (0 K), 26.88^b^ (0 K), 25.26^c^ (100 K), 26.09^d^ 25.21^c^ (300 K), 26.08^d^ (300 K), 26.03^h^ (100 K), 25.95^h^ (300 K)	12.78	9.20	16.0^a^ (0 K), 19.0^a^ 11.95^f^ 15.76^d^ (0 K), 8.70^b^ (0 K), 19.37^c^ (100 K), 16.66^c^ (300 K), 13.3^d^ (300 K), 12.3^c^ (300 K), 14.34^e^ (300 K), 13.17^h^ (100 K), 8.97^h^ (300 K)
IRMOF-10	34.28	34.69	34.59	35.35^b^ (0 K), 34.82^d^ (100 K), 34.48^d^ (300 K), 34.37^e^ (300 K), 34.73^h^ (100 K), 34.62^h^ (300 K)	5.20	3.51	9.2^d^ (0 K), 8.25^f^ (0 K), 6.00^b^ (0 K), 3.5^d^ (300 K), 7.41^e^ (300 K), 5^g^ (300 K), 6.95^h^ (100 K), 5.23^h^ (300 K)

a From ref (36).

b From ref (72).

c From ref (73).

d From ref (73).

e From ref (74).

f From ref (75).

g From ref (19).

h From ref (59).

### Gas Adsorption Profile Accounting for the Adsorbate Effect

**Figure 2:**
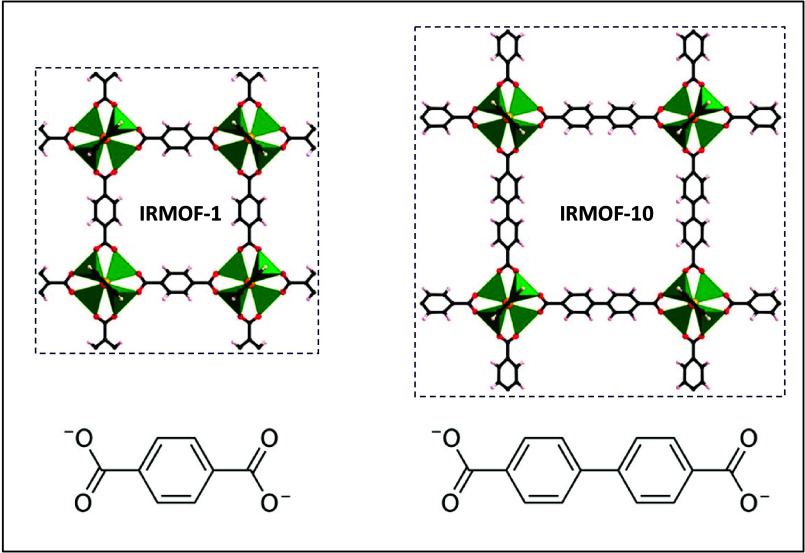
Variations in the types of organic linkers incorporated into the IRMOF-1 and IRMOF-10 structures.

The HDNNPs developed and tested include all IRMOF-*n* (*n* = 1, 4, 6, 10, or 16). In principle, one could perform simulations for all of these MOFs. In our investigation, we have specifically selected two materials, IRMOF-1, and IRMOF-10, extended via benzenedicarboxylate and biphenyldicarboxylate linkers, respectively. IRMOF-1, with its shorter linker, is comparatively more rigid than IRMOF-10. The choice of these chemically similar linkers, depicted in Figure 2, enables a systematic exploration of the impact of pore size. In addition, we aimed to compare the effect of the adsorbate during gas adsorption by using gases such as H_2_ and CH_4_ that quite vary in size from each other in our simulations.

**Figure 3:**
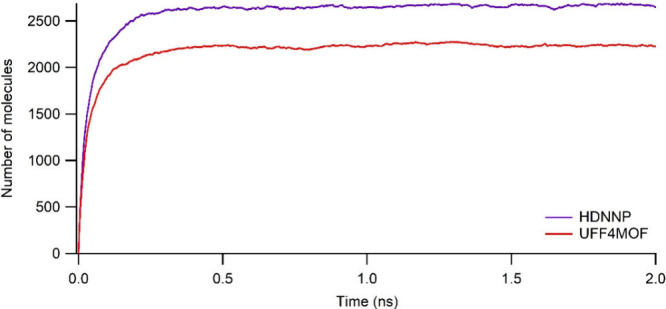
Number of adsorbed H_2_ molecules vs time for the simulations of adsorption of RMOF-1 for hydrogen gas at 100 bar and 77 K using HDNNP (blue) and UFF4MOF (red) for MOF definition during adsorption–relaxation simulations.

To demonstrate the success of our HDNNP for accurately depicting the structural characteristics of MOFs in comparison to classical force fields (FFs), we investigated the gas adsorption profiles of IRMOF-1 and IRMOF-10 structures with different pore sizes but an identical overall topology. Our approach involved calculations utilizing a combination of HDNNP/classical FF potentials and GCMC/MD combined simulations leading to adsorption–relaxation flexibility. Figure 3 shows the H_2_ gas uptake of IRMOF-1 at 100 bar and 77 K using the adsorption–relaxation model based on the UFF4MOF and HDNNP force field in the definition of MOF. The number of H_2_ molecules loaded on IRMOF-1 reaches a plateau at around 250 ps in both force fields. Thereafter, it maintains its saturated concentration of ∼2200 molecules in the case of UFF4MOF. This figure illustrates the convergence of the GCMC/MD simulation at one specific pressure (100 bar) rather than an adsorption isotherm. The adsorption isotherm is created by the collection of different MD simulations at all pressure values (1–100 bar). Although the simulations are converged within 250 ps, we used the average loading of the last 1 ns to ensure the converged loadings. Since the process is involved in both MD and GCMC simulations, at each insertion of gas molecules, the system is equilibrated at the preset pressure. Allowing the system to relax during adsorption causes MOF structures to realign themselves with this new condition, in which adsorbate molecules are inserted or deleted.

Defining the MOF structure with HDNNP instead of UFF4MOF, hydrogen gas loading was monitored throughout the MD/GCMC simulation at 100 bar and 77 K (Figure 3 and Figure S11). Notably, saturation achieved by loading more H_2_ molecules (∼2600) than with UFF4MOF (∼2200) suggests that the HDNNP approach captures adsorption–relaxation processes in a distinct manner. Like UFF4MOF, the same plateau is reached and steady loadings are maintained with HDNNP at around 250 ps, showing the convergence of the MD/GCMC simulations. The loading in Figure 3 corresponds to a single pressure value of 100 bar (i.e., one point in the adsorption isotherm), and the adsorption isotherm is created by the collection of plateaus of several MD simulations at several pressure values (1–100 bar).

**Figure 4:**
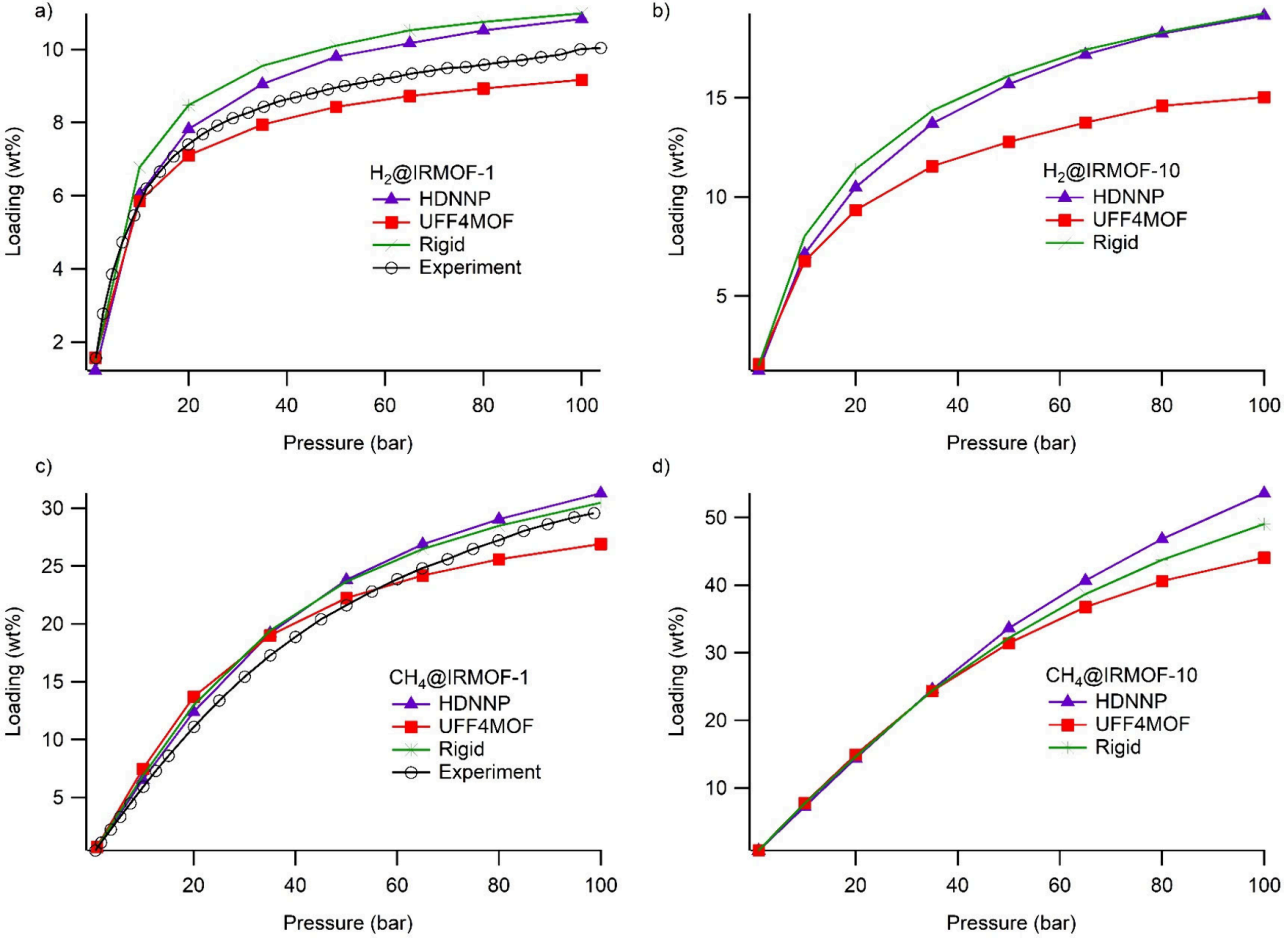
Adsorption profiles showing the uptake using HDNNP potentials (blue) and UFF4MOF simulations (red) for MOF definition along with rigid definition (green) and experimental (black) data: (a) H_2_ on IRMOF-1 at 77 K, (b) H_2_ on IRMOF-10 at 77 K, (c) CH_4_ on IRMOF-1 at 300 K, and (d) CH_4_ on IRMOF-10 at 300 K.

By repeating these simulations for several pressure values, we generated adsorption isotherms. Figure 4 shows H_2_ and CH_4_ adsorption isotherms on IRMOF-1 and IRMOF-10 obtained when the MOF is defined by either HDNNP or UFF4MOF as well as rigid definition of the MOF structure and experimental data.^76^ At low pressures (up to 20 bar), the general behavior of both methods (HDNNP vs UFF4MOF) and the rigid definition agree with each other and experimental values. However, when the pressure is increased, HDNNP tends to slightly overestimate the experimental values. Similarly, a rigid definition also predicts higher values than the experimental loadings. This should be because simulations employ impurity-free and perfect structures, as opposed to experiments, which are affected by impurities.

In the case of H_2_ adsorption on IRMOF-1, which have been well studied both experimentally and computationally, the rigid definition and HDNNP follow similar trends, in particular, at higher pressure values, both of which overestimate the experimental results. However, at higher pressures, UFF4MOF underestimates H_2_ uptake. Since experimental results are between the UFF4MOF-predicted and HDNNP-predicted values, it is hard to draw a conclusive picture from this alone. Apparently, H_2_ molecules have little impact on the IRMOF-1 structure, likely due to their weak interactions with rather rigid IRMOF-1 with large pore sizes. However, one could infer that HDNNP could successfully reflect this rigidity of this MOF during H_2_ adsorption, while the UFF4MOF prediction deviates slightly from the rigid definition.

**Figure 5:**
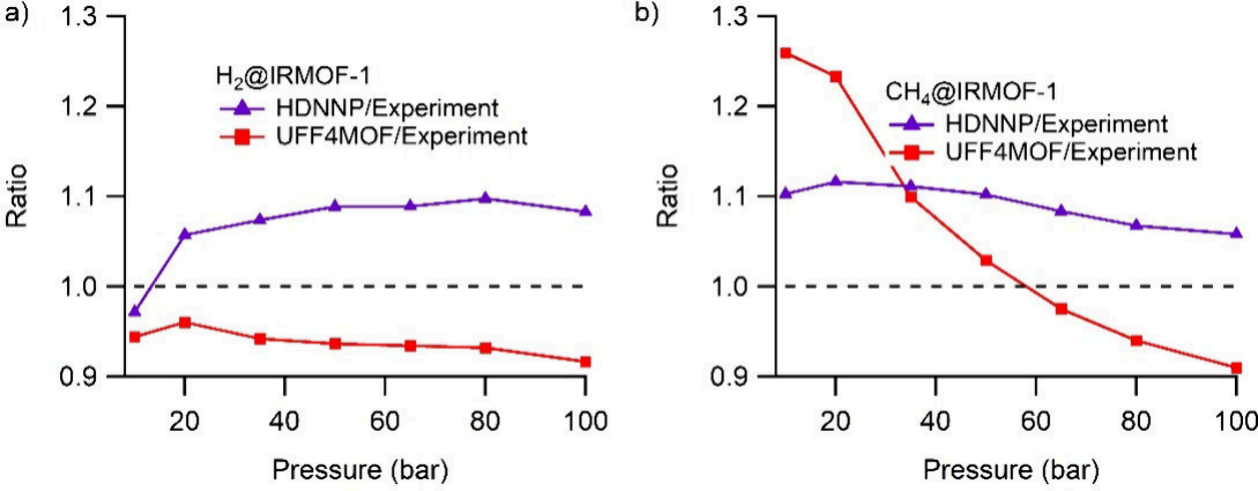
Predicted/experimental loading ratios at different pressures. Predicted values are for UFF4MOF and HDNNP, showing a steady constant line over the pressures in the case of HDNNP while that of UFF4MOF is divergent.

In the case of adsorption of CH_4_ on IRMOF-1, rigid and HDNNP definitions are in perfect agreement with experimental results specifically under higher-pressure conditions, whereas UFF4MOF deviates from experimental results. The same overall conclusion with H_2_ uptakes could be drawn for methane adsorption predicted by the simulations using HDNNP versus UFF4MOF for IRMOF-1. For this adsorbate, both HDNNP and UFF4MOF correctly predict the experimental values at low pressures. The trend of CH_4_ uptake over different pressures totally diverged from the experiment in UFF4MOF, whereas for HDNNP, it is still in good agreement with experimental values. This divergence of UFF4MOF is also reflected in the void fraction and pore volume values (Figures S12 and S13). A small deviation as in the case of H_2_ adsorption can easily be fixed by setting an offset, which might be due to impurities^77^ or quantum effects.^78^ For a better representation of how the UFF4MOF-based calculation diverges from experiment while HDNNP follows the experiment, we calculated the ratio of predicted values (from both methods) to experimental values (Figure 5). The of HDNNP/experimental ratio follows a rather steady constant line at 1.1 at all pressures, while the UFF4MOF/experimental ratio does not have a constant value over the pressures and changes at every different pressure in particular for CH_4_@IRMOF-1. From these observations, we can conclude that the high-pressure gas uptakes of MOFs are more accurately modeled by HDNNPs.

Figure 4 also shows hydrogen and methane adsorptions on IRMOF-10. Unfortunately, there are no experimental data on the adsorption of these gases in IRMOF-10 in the literature. Thus, deciding whether UFF4MOF or HDNNP predicts the gas uptakes more accurately is not trivial. IRMOF-10 owing to its longer linker than IRMOF-1 tends to behave differently for gas adsorption. Interestingly, the HDNNP-predicted adsorption profile on IRMOF-10 is quite similar to that of the rigid model. In particular, the H_2_ adsorption profiles of both MOFs are quite similar to the rigid model, whereas UFF4MOF dramatically underestimates the loadings. On the other hand, the HDNNP, UFF4MOF, and rigid models differ from each other at the extreme pressures (near 100 bar) for methane adsorption on IRMOF-10. This might be due to IRMOF-10 having longer linker to behave slightly less rigidly at high pressures for larger gas molecules.

Table 2 lists the absolute values of uptakes (wt %) of H_2_ and CH_4_ gases in IRMOF-1 and IRMOF-10 at 100 bar. The choice of comparison at this pressure is to show how the UFF4MOF can fail when extreme conditions (such as higher pressures) are reached. Furthermore, when the adsorbate becomes larger, UFF4MOF diverges significantly from that of NNP. The absolute value of CH_4_ uptake predicted by UFF4MOF deviates by 2.67 from the experimental value, while that of HDNNP is only 1.71. On the other hand, for CH_4_ uptake on IRMOF-10, one can totally mispredict the real adsorption amount. Although there are no experimental data for CH_4_ uptake on IRMOF-10, the NNP-based adsorption value reaches 53.59% while UFF4MOF predicts a value of 44.06%. Because of the HDNNP-driven GCMC/MD simulations, which are hindered otherwise with UFF4MOF, the real CH_4_ uptake capacity of IRMOF-10 under extreme conditions is revealed, noting that this finding should be verified further by experiments.

**Table 2:** Total H_2_ and CH_4_ Uptake on IRMOF-1 and IRMOF-10 (wt %) at 100 Bar and 77 K for H_2_ and 300 K for CH_4_^a^

	IRMOF-1		IRMOF-10	
	H_2_	CH_4_	H_2_	CH_4_
UFF4MOF	9.16 (0.08)	26.90 (0.47)	15.02 (0.08)	44.06 (0.71)
HDNNP	10.83 (0.08)	31.28 (0.50)	19.14 (0.17)	53.59 (0.79)
experiment	10.01	29.57	–	–

a The numbers in parentheses are standard deviations.

The nature of this unusual feature of IRMOF-10 for CH_4_ uptake under the extreme conditions could be attributed to activation of the low-lying vibrations (transverse vibrations, breathing, and collective motions) of the organic linker in IRMOF-10, which are not active under non-extreme conditions. These motions are apparently better represented in HDNNP-based simulations than in UFF4MOF-based ones.

### On-the-Fly Structural Analysis

One of the most prominent features of adsorption–relaxation simulations (i.e., GCMC/MD combined technique) is that it allows one to monitor structural changes dynamically occurring during gas adsorption on MOFs. The current computational technology does not allow GCMC simulations combined with ab initio molecular dynamics simulations for such bulky systems. On the other hand, our HDNNP can provide sufficiently accurate results in terms of the dynamic changes during gas adsorption. Here, as a case study, we show methane adsorption on IRMOF-10 at 300 K and 100 bar. Our choice of these adsorbent–adsorbate pair and extreme conditions is to clearly reveal how flexibility and/or rigidity can be better represented by HDNNP rather than classical force field, UFF4MOF.

**Figure 6:**
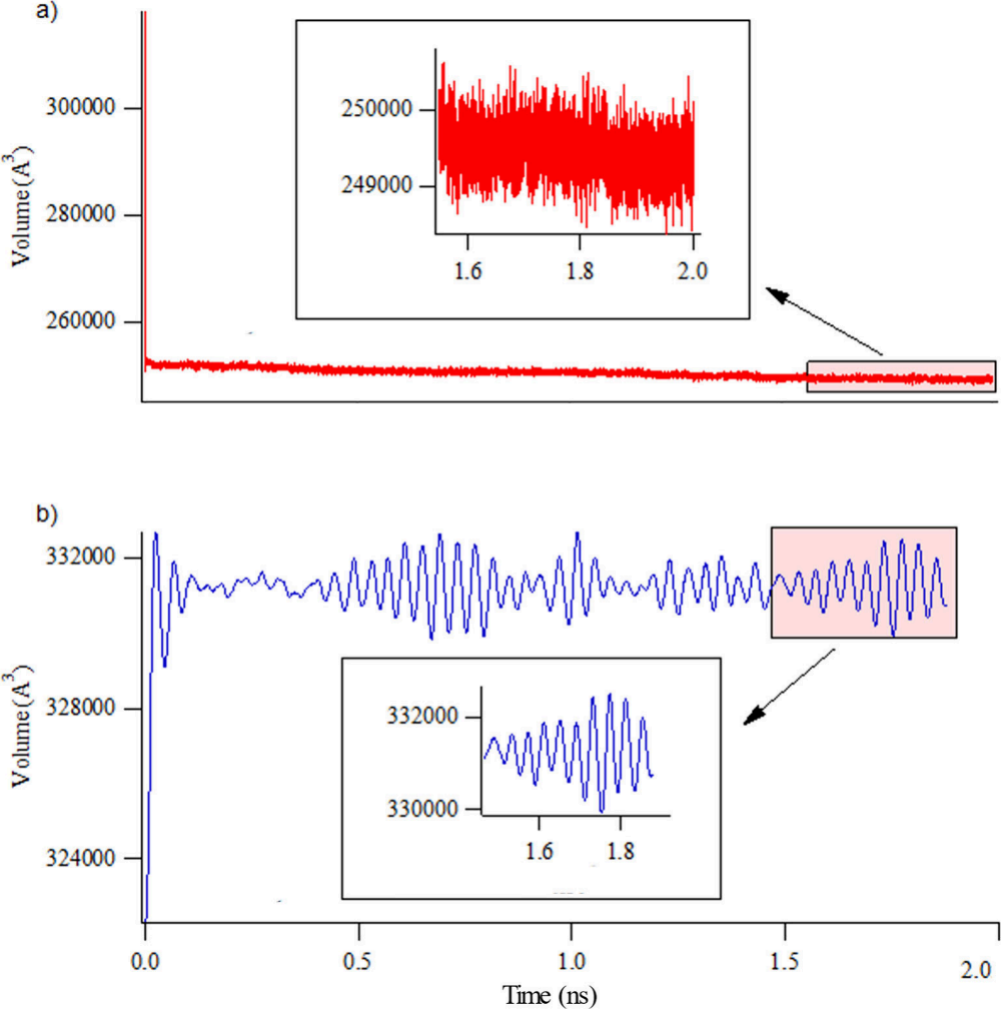
Total volume change during adsorption–relaxation simulations of CH_4_ on IRMOF-10 using (a) UFF4MOF and (b) HDNNP for the definition of the MOF structure.

The main reason for the capacity difference between HDNNP and UFF4MOF is the misprediction of the total volume (and thus pore volume) by the latter, as a result of the limitation of the classical definition. Figure 6 shows the total volume of the equilibrated system for both methods during the last 500 ps of 2 ns long GCMC/MD simulations. One of the most important characteristics of the volume change throughout the simulation is that the averaged ensemble value of the volume is much larger in the case of HDNNP (∼331 250 Å^3^) than UFF4MOF predictions (∼249 750 Å^3^) in a 2 × 2 × 2 supercell of IRMOF-10. Another characteristic difference between the UFF4MOF and HDNNP definitions is the fluctuation of the volume at each MD step. In the case of UFF4MOF, the values oscillate in a random fashion at 2000 Å^3^. This is most likely a limitation of the classical definition of the force field, in which at individual time steps, the molecules can visit unphysical configurations. In the case of HDNNP, however, much smoother and organized fluctuation occurs. The intensity of the fluctuation reaches a maximum at 1750 ps, which might indicate correlated motions of the MOF structure such as breathing, a typical experimental feature of MOFs.

**Figure 7:**
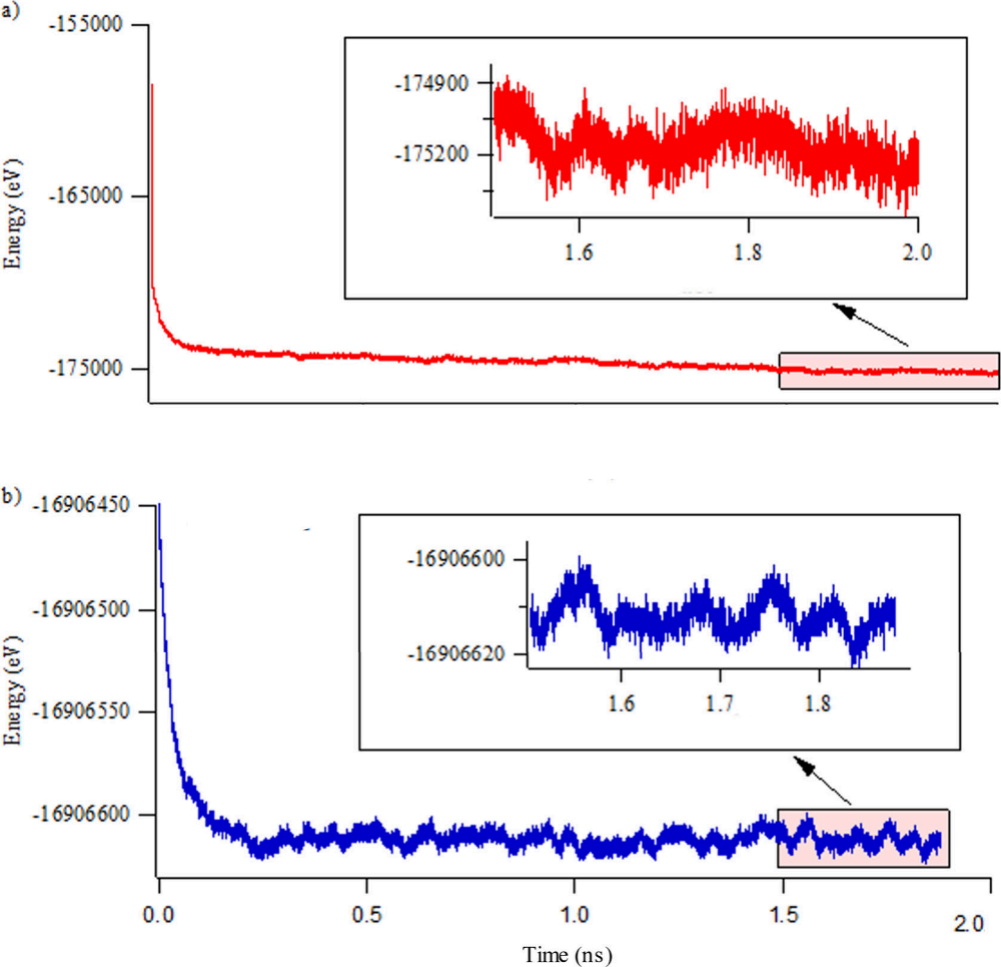
Total potential energy during adsorption–relaxation simulations of CH_4_ on IRMOF-10 using (a) UFF4MOF and (b) HDNNP for the definition of the MOF structure.

This random fluctuation in the UFF4MOF definition also occurs in the total energies of the system. In the classical FF definition of the MOF structures, the bonds are generally constrained to harmonic motions during the simulations. This keeps the structure whole when large energy jumps occur during the simulation. Figure 7 shows the total potential energy throughout the trajectory of the last 500 ps of a 2 ns long GCMC/MD simulation. In the case of UFF4MOF, the energy oscillates by 500 eV (between 174 900 and 175 400 eV) whereas this oscillation is only 20 eV in the case of HDNNP, which is much more physical considering the system consists of thousands of atoms.

These instantaneous energy increases in the UFF4MOF definition might lead to false predictions of the porosity of the structure, which is ultimately responsible for gas adsorption capacities. In order to investigate the consequences of these large energy fluctuations in the case of UFF4MOF, we have also monitored important distances, angles, and dihedrals in the MOF structures.

**Figure 8:**
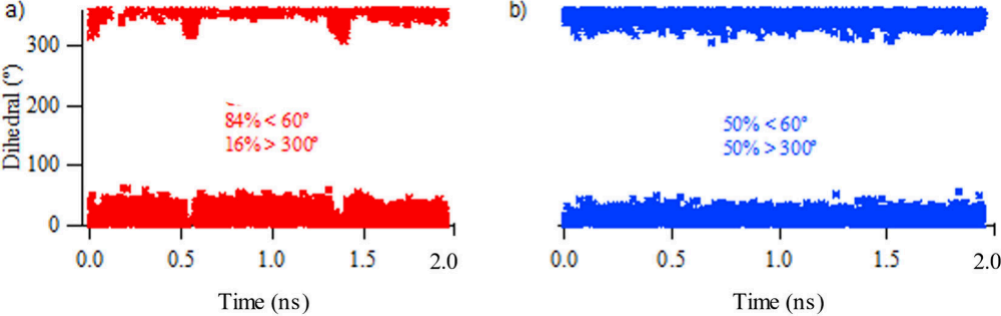
Dihedral angles and populations (percentages in the insets) for carboxyl oxygen atoms and the phenyl rings during adsorption–relaxation simulations of CH_4_ on IRMOF-10 for (a) UFF4MOF and (b) HDNNP.

**Figure 9:**
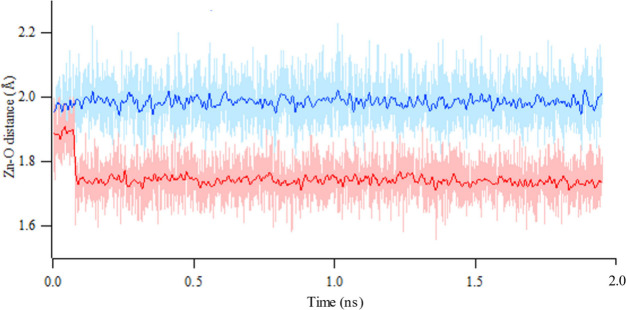
Smoothed (bold) and unsmoothed (faint) distance between Zn and carboxyl O atoms during adsorption–relaxation simulations of CH_4_ on IRMOF-10 for UFF4MOF (red) and HDNNP (blue).

Figure 8 shows the dihedral angle between the planes of the carboxyl group and phenyl rings, O_carboxyl_–C_carboxyl_–C_Ph_–C_Ph_. The dihedral angles are populated in two distinct regions, one being in the range of 0–60° referring to the positive direction of the torsional motion and the other in the range of 300–360° referring to the negative direction. From this analysis, it could be inferred that UFF4MOF does not correctly reflect this torsional vibration and predicts that the angle is most populated at 60°. This slight angle fictitiously brings the Zn and O atoms closer (to a distance of 1.75 Å) than the experimental distance of 1.95 Å (Figure 9). This eventually causes the MOF structure to shrink and to have a smaller volume than expected. This picture is more obvious in the pore volume and void fraction changes throughout the MD simulation. On the other hand, HDNNP reflects the torsional vibration in a perfect distribution with a population of 50% for each direction.

Another perspective of mechanistic analysis during the adsorption process is monitoring the radial distribution functions (RDFs). Radial distribution functions provide the probabilities of finding a particle within a cutoff distance and directly correlated with the X-ray diffraction pattern of the crystalline materials. In principle, the most detailed structural information, such as how the MOF structure changes while the guest molecules are loading, where the gas molecules localize in the MOF structure, etc., can be obtained. However, experimentally analyzing RDF plots of MOFs during the adsorption process for such mechanistic details is an incredibly challenging task. On the other hand, adsorption–relaxation simulations allow us to generate RDF plots and thus determine how the MOF structure behaves according to the external condition and guest molecule adsorption.

**Figure 10:**
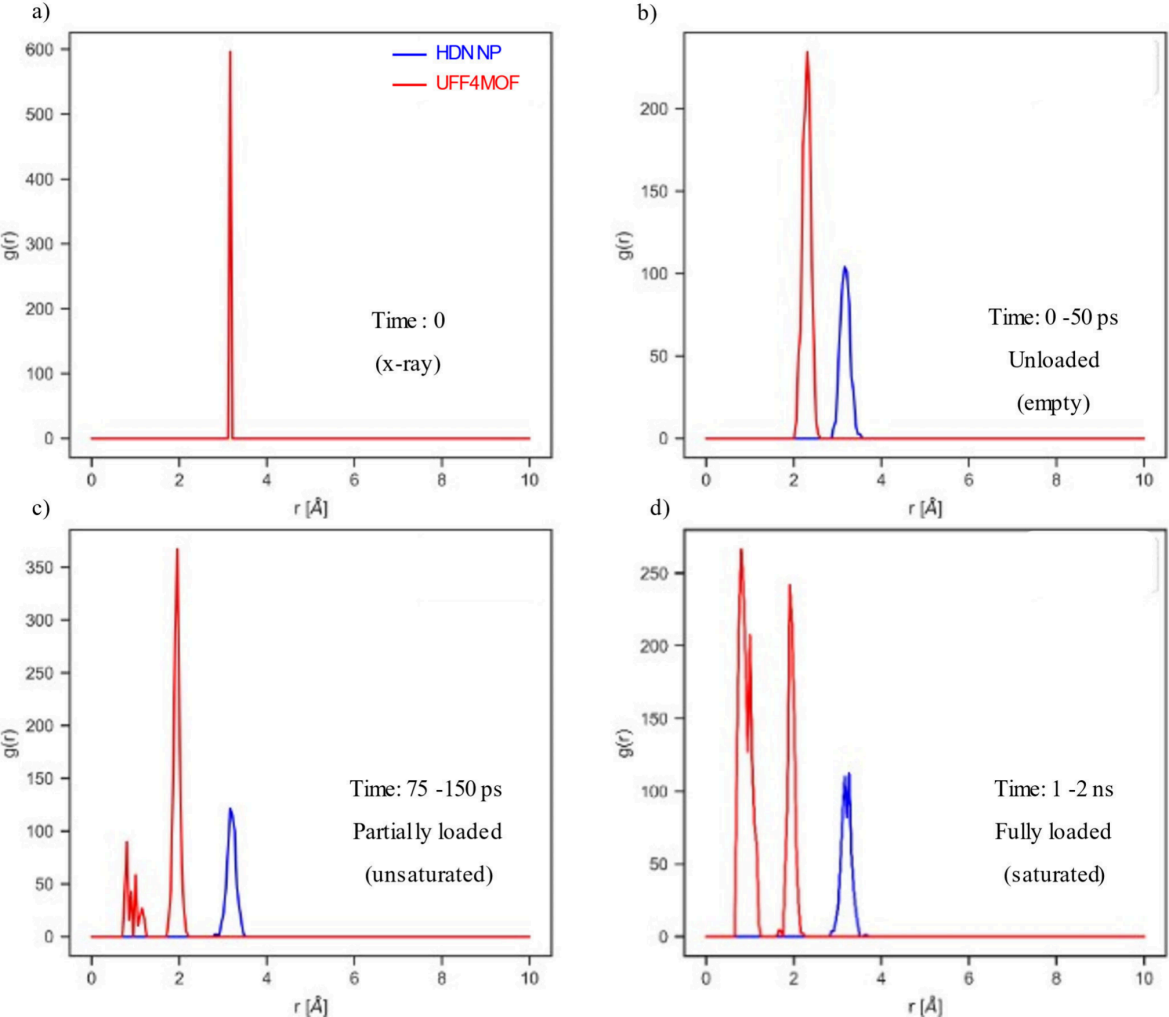
Radial distribution function for the Zn–Zn atom pair during adsorption–relaxation simulations of CH_4_ on IRMOF-10 at 300 K and 100 atm.

Here, we investigated the RDF of IRMOF-10 while adsorbing on the fly methane gases by UFF4MOF and HDNNP description of the MOF structure in the GCMC/MD simulations. Since there are several different atom types in this MOF structure, numerous RDF plots can be generated. We calculated the RDF values, *g*(*r*), shown in the figure by averaging the distances between atom pairs over the entire simulation cell and across the simulation time period, thereby reflecting the number density rather than a single time frame. Figure 10 shows the most interesting RDF plot for the Zn–Zn pair of IRMOF-10 during the methane adsorption process at 300 K and 100 atm. Initially (at time zero), the MOF structure has a sharp peak at 3.2 Å, which shows a perfect alignment of Zn atoms throughout the 2 × 2 × 2 supercell. When the GCMC/MD simulations started but no methane molecules are yet adsorbed (0–50 ps), the MOF structure starts deviating from the crystal structure due to the external field (pressure and temperature) and dynamic motions defined by either UFF4MOF or HDNNP. According to the UFF4MOF definition of the MOF structure, the distance between the Zn pairs uniformly decreases to 2.3 Å, which shows the shrinkage of the MOF structure. Having Zn atoms at such a small distance is not physical, clearly indicating the failure of UFF4MOF. On the other hand, in the case of the HDNNP definition of the MOF structure, this uniform distance does not shift but rather broadens during these time frames. Broadening occurs due to Zn-involved vibrations. When the methane molecules are loaded either partially at 75–100 ps or fully at 1–2 ns, the UFF4MOF-predicted structure shows that Zn atoms form two different coordination shells belonging to other Zn atoms. The first is located at 2 Å, and the second is around 1 Å. Therefore, according to UFF4MOF prediction, Zn atoms are no longer well organized, indicating the disrupted MOF structure or complete failure of the UFF4MOF definition of Zn atoms. This is the opposite of what is seen in HDNNP predictions. Methane adsorption in the HDNNP representation of the MOF does not damage the MOF structure. This is a very striking result for two reasons. The first is that UFF4MOF is a widely used force field in the literature for GCMC/MD simulations, and it can totally fail the correct modeling and/or simulation of the system at these time scales. Second, the IRMOF-10 structure can be very promising in reversible applications of methane adsorption.

**Figure 11:**
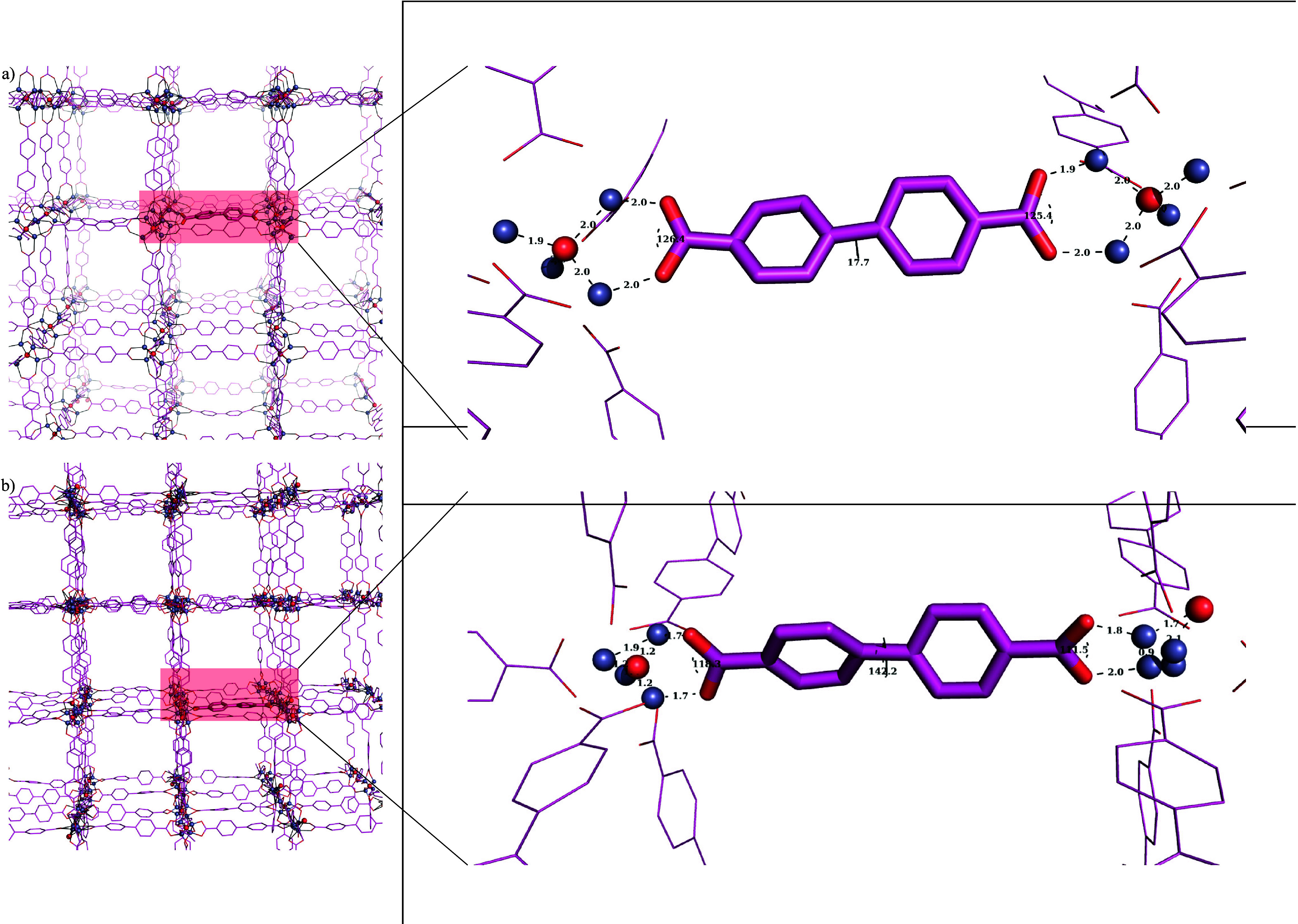
Frame near the end of the MD simulation of CH_4_ adsorption on IRMOF-10 illustrating Zn_4_–O, the organic linker, and important distances and angles in (a) UFF4MOF and (b) HDNNP, clearly indicating the failure of UFF4MOF. Guest molecules and hydrogen atoms have been omitted for the sake of clarity.

In order to understand why UFF4MOF-defined MD simulations show abnormal Zn–Zn RDF plots, we extracted several snapshots from the simulations. Figure 11 compares the MOF structures near the end of the GCMC/MD simulations of CH_4_@IRMOF-10 with both UFF4MOF and HDNNP definitions. In the case of the UFF4MOF definition, the coordination of Zn atoms around the inorganic O atom becomes non-uniform and some of these O atoms escape from the Zn coordination shell. This results in Zn_4_ clusters shrinking to ∼1 Å, distorting the node. This distortion increases with simulation time. Distortion of the nodes and shrinkage of Zn_4_ clusters in UFF4MOF due to the escape of inorganic O atoms from the coordination shell also cause the angle of the carboxylic group (OĈO) to decrease from 118° to 111°. On the other hand, in the case of HDNNP, the coordination around the inorganic O atom is uniformly maintained throughout the simulations. Thus, the Zn–Zn distance and the angle of the carboxylic group (OĈO) stay at 1.9–2.0 Å and ∼125°, respectively, with a slight change due to vibrations. Similar to the angle of the carboxylic group, the dihedral angle between the two phenyl rings is also characteristic for HDNNP and UFF4MOF. In the case of HDNNP, the two aromatic rings have a slightly smaller dihedral angle (∼18°), maintaining the aromaticity (by maintaining planarity), whereas in the case of UFF4MOF, these rings have larger angle values (being more perpendicular) of 140° (tilted by 50°). It should be noted that these angles and distances play a particularly vital role in the low-frequency vibrations of the MOF structure, which has been shown to be responsible for the MOFs’ other interesting features such as breathing and negative thermal expansion.^59,79^

To determine whether the distortion is unique to UFF4MOF or a common issue across all classical force fields, we also conducted test simulations using UFF for the MOF structure instead of UFF4MOF (data not shown). The distorted Zn centers appear only with UFF4MOF. However, with UFF, the angles of the carboxylic groups and the dihedral angle between the phenyl rings remain similar to those in UFF4MOF, at 110–116° and 140°, respectively, resulting in lower adsorption values than those predicted by HDNNP.

Other than Zn–Zn, we have also explored the distribution of O–O and C–C atoms (Figure S14) within the MOF along with the CH_4_–CH_4_ distribution. The O–O RDF plots show a result similar to that of the Zn–Zn RDF plots in reflecting damaged Zn_4_O in the MOF structure by the UFF4MOF definition. The C–C distributions in MOF structure distribution plots are almost identical in the cases of UFF4MOF and HDNNP definitions. This clearly shows the success of UFF4MOF in covalent bonds. Similarly, CH_4_–CH_4_ distributions in both definitions are identical and reflect the Lennard-Jones distances as the HDNNP only defines the MOF structure (i.e., not the gas molecules).

**Figure 12:**
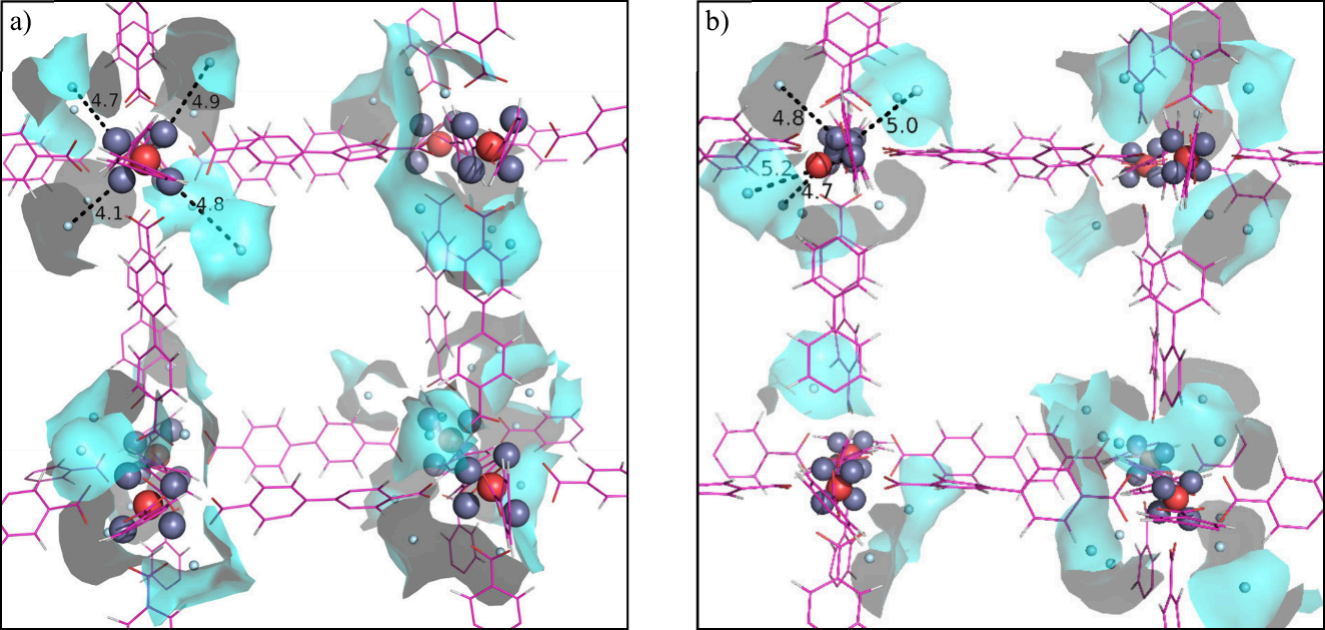
Displacement of methane molecules within 5 Å of Zn atoms (secondary shell) in (a) HDNNP and (b) UFF4MOF.

We also explored the distribution of the loaded gas within the MOF structure. When the loading is completed and sufficient MD simulations are run, the gas molecules cover all of the accessible volumes. However, the gas molecules around the nodes are denser. Figure 12 shows the loaded molecules in the proximity of Zn atoms. Since the first coordination shell of the Zn atoms is fully occupied, the gas molecules are loaded into the second solvation shell created by the two adjacent linkers. In the case of HDNNP, there are 35 methane molecules within 5 Å of Zn atoms in the unit cell. The methane molecules are distributed uniformly in these secondary shells, with a maximum distance of 4.9 Å. On the other hand, in the case of UFF4MOF, there are only 29 methane molecules for the same space. In particular, the damaged Zn_4_O nodes include fewer methane molecules around them due to the larger distance (5.2 Å) between Zn and methane molecules.

## Conclusion

In this study, we have trained our high-dimensional neural network potential (HDNNP) to describe rotationally and translationally invariant energies and forces of IRMOFs at the DFT level of accuracy using the fragmentation technique to study H_2_ and CH_4_ adsorption isotherms considering flexibility. Then, our HDNNP was successfully utilized to study the flexibility of MOFs during gas adsorption by means of an “adsorption–relaxation” model in which MD and GCMC simulations were performed simultaneously. Our results showed that the classical FF may diverge from experiments when accounting for flexibility in MOFs whereas our HDNNP follows a much better trend with respect to experimental values. In addition, in the absence of experiments, data showed that the real number of gas uptake values can be more than what classical FF predicts. The study highlighted the importance of considering MOF flexibility in gas adsorption simulations and indicated the potential for higher gas uptake values than those predicted by traditional FF models. In particular, other MOFs with more flexibility involved will benefit from the findings of the current studies. The studies will open gates for the studies about MOF behaviors such as expanding, contracting, and breathing effects as well as phase transitions and diffusion rates of different gases. In addition to its findings among the MOFs studied, since these studies are among the first studies in the field, it sets a step-by-step guide for the reader to conduct such ultra-modern simulations.

## Supporting Information Available

Supplementary figures of IRMOF-*n* (*n* = 1, 4, 6, 7, or 10), training and loss curves, histogram of energy errors, DFT- and NNP-produced energies of fragments, gas loadings for IRMOF-1 and IRMOF-10, radial distribution function plots for IRMOF-10, and LJ potential parameters for gases and frameworks (PDF)
