# Supplementary material for: Modeling Gas Adsorption and Mechanistic Insights into Flexibility in Isoreticular Metal–Organic Frameworks Using High-Dimensional Neural Network Potentials
Source: Langmuir. 2025 Mar 14;41(11):7323–35. doi: 10.1021/acs.langmuir.4c04578 (PMC11948474; doi:10.1021/acs.langmuir.4c04578)
Supplement: Supplementary file 1 — la4c04578_si_001.pdf [file la4c04578_si_001.pdf]

Modeling Gas Adsorption and Mechanistic Insights into Flexibility in Isoreticular Metal–Organic Frameworks using High-Dimensional Neural Network Potentials

Omer Tayfuroglu[1], Abdulkadir Kocak, Yunus Zorlu

Department of Chemistry, Gebze Technical University, 41400 Gebze, Kocaeli, Turkey

Validation/test of HDNNPs

The dataset was split into training (~89k, 90%) and test (~9k, 10%) sets. Hyper parameters were used as default values except that we used 1:15 ratio of energy to force updates. We stopped the training after 60 epochs (although it was converged within 30, **Figure S2**).

The RMSE of energy and forces are 0.0017 eV/atom and 0.23 eV/Å, respectively. The distribution of the errors is random and fits to a normal Gaussian function (**Figure S3**). For all the fragments, NNP produced energies and forces are in a complete agreement with DFT calculated values (R^2≈0.999-1.000, **Figure S4** and **Figure S5**). In addition, we analyzed the absolute errors produced by NNP and DFT calculations for the fragments. We observed that the absolute errors on almost all of the fragments lie within the narrow range of 0.005 eV/atom and 0.5 eV/Å for energy (**Figure S6**) and force (**Figure S7**), respectively.

[1] Corresponding Author

E-mail: otayfuroglu@gtu.edu.tr Phone:+902626053083

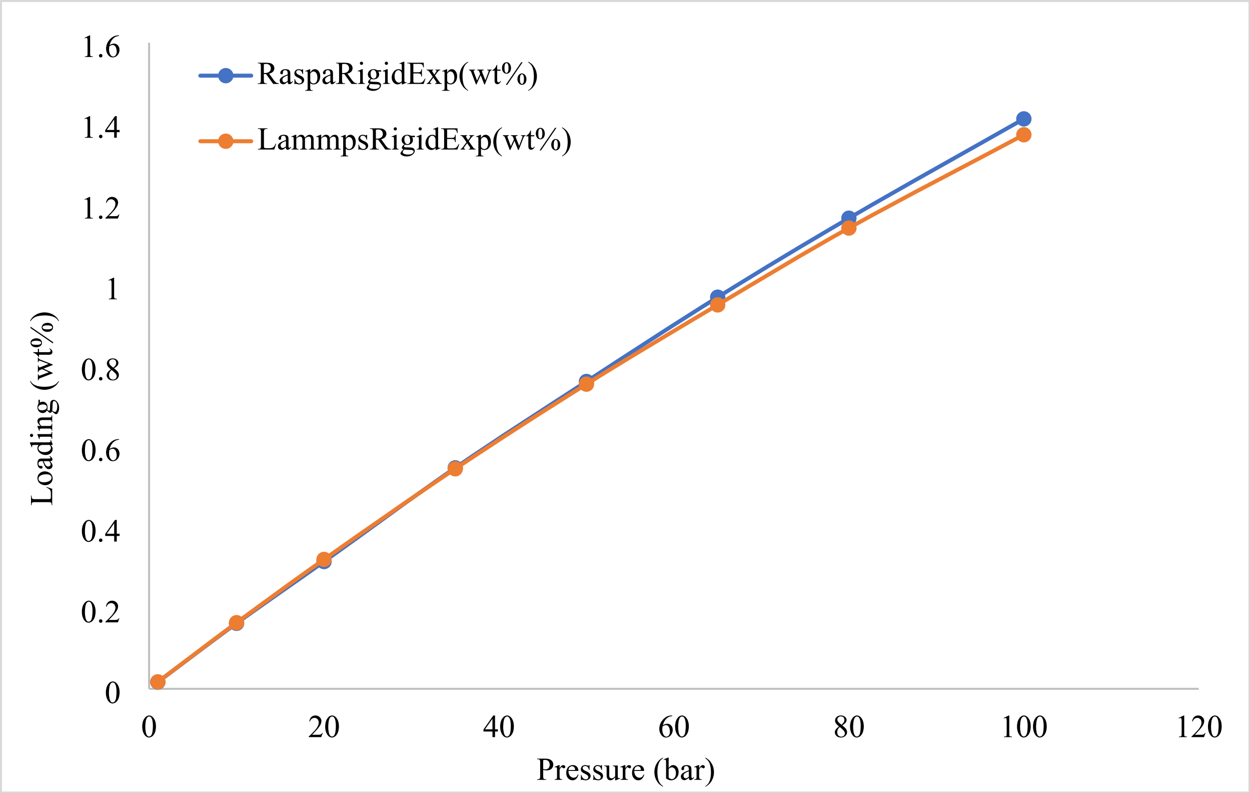

Figure S1: H2 uptakes calculated by GCMC simulations at 298K using RASPA (blue) and LAMMPS (orange).

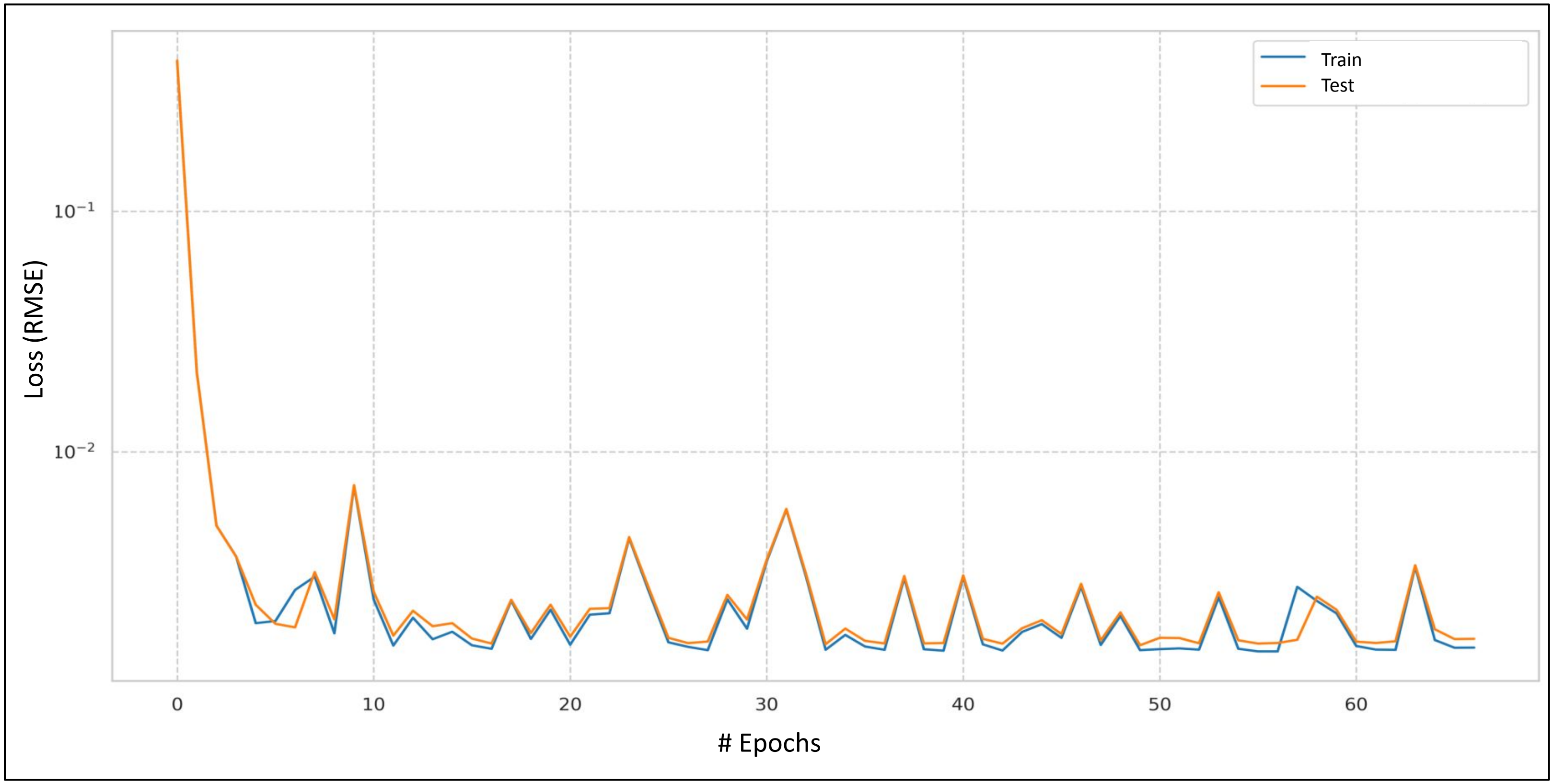

Figure S2 Training loss curves of single HDNNP for IRMOFs.

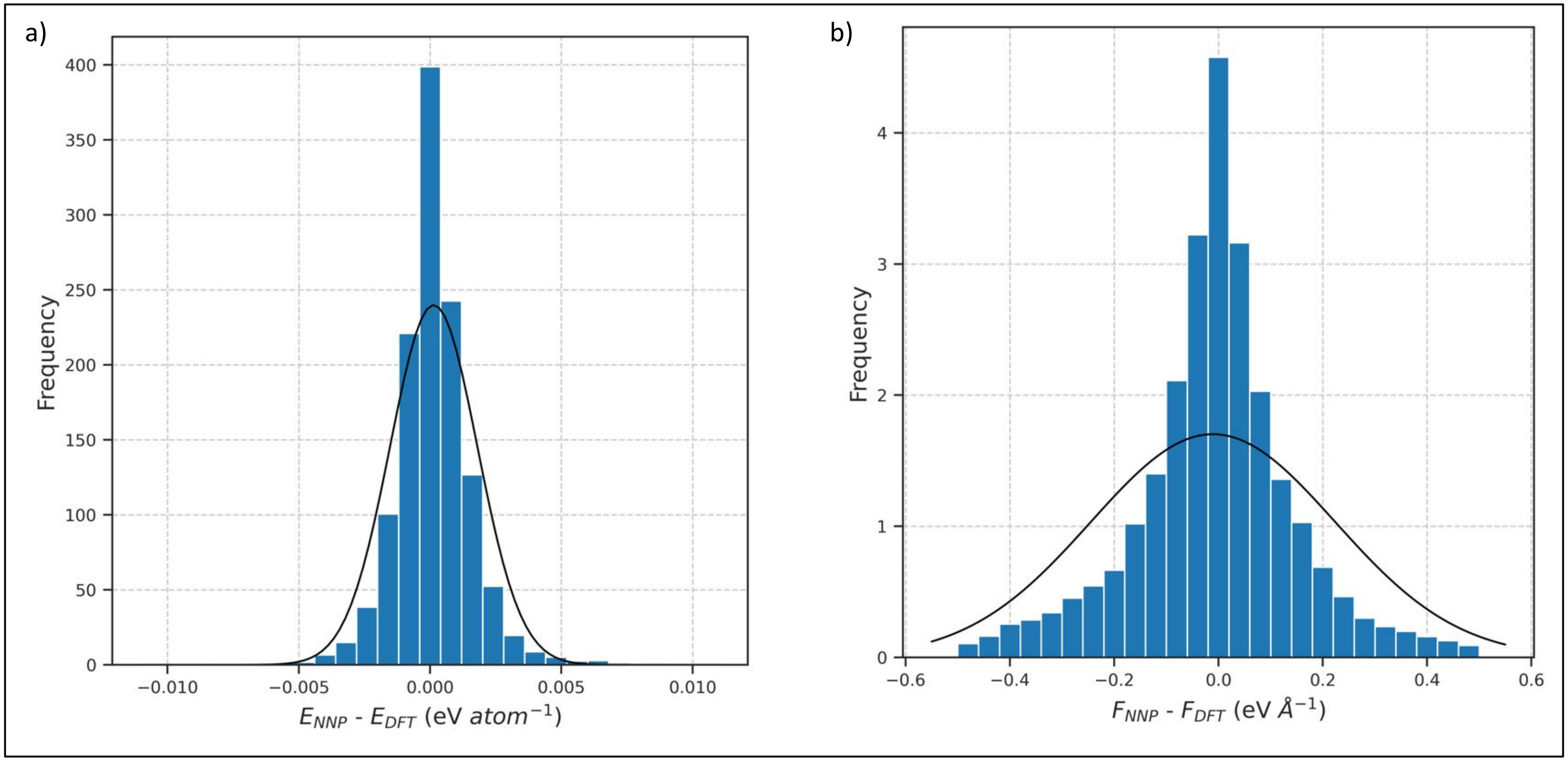

Figure S3 Histograms of energy errors show normal distribution a) training b) test.

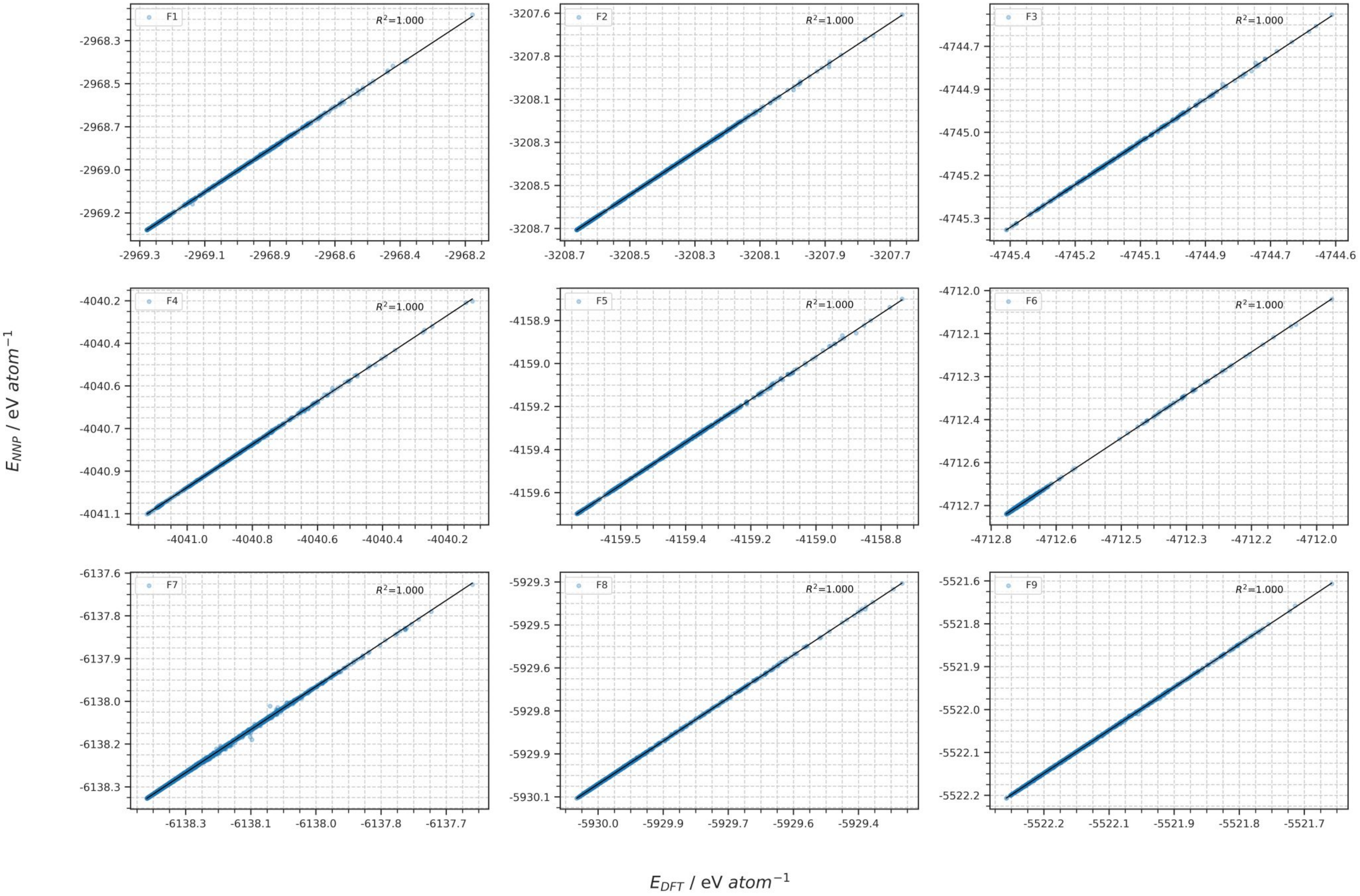

Figure S4 Correlation of between the DFT energy and the predictions of the NNP model energy based on test set.

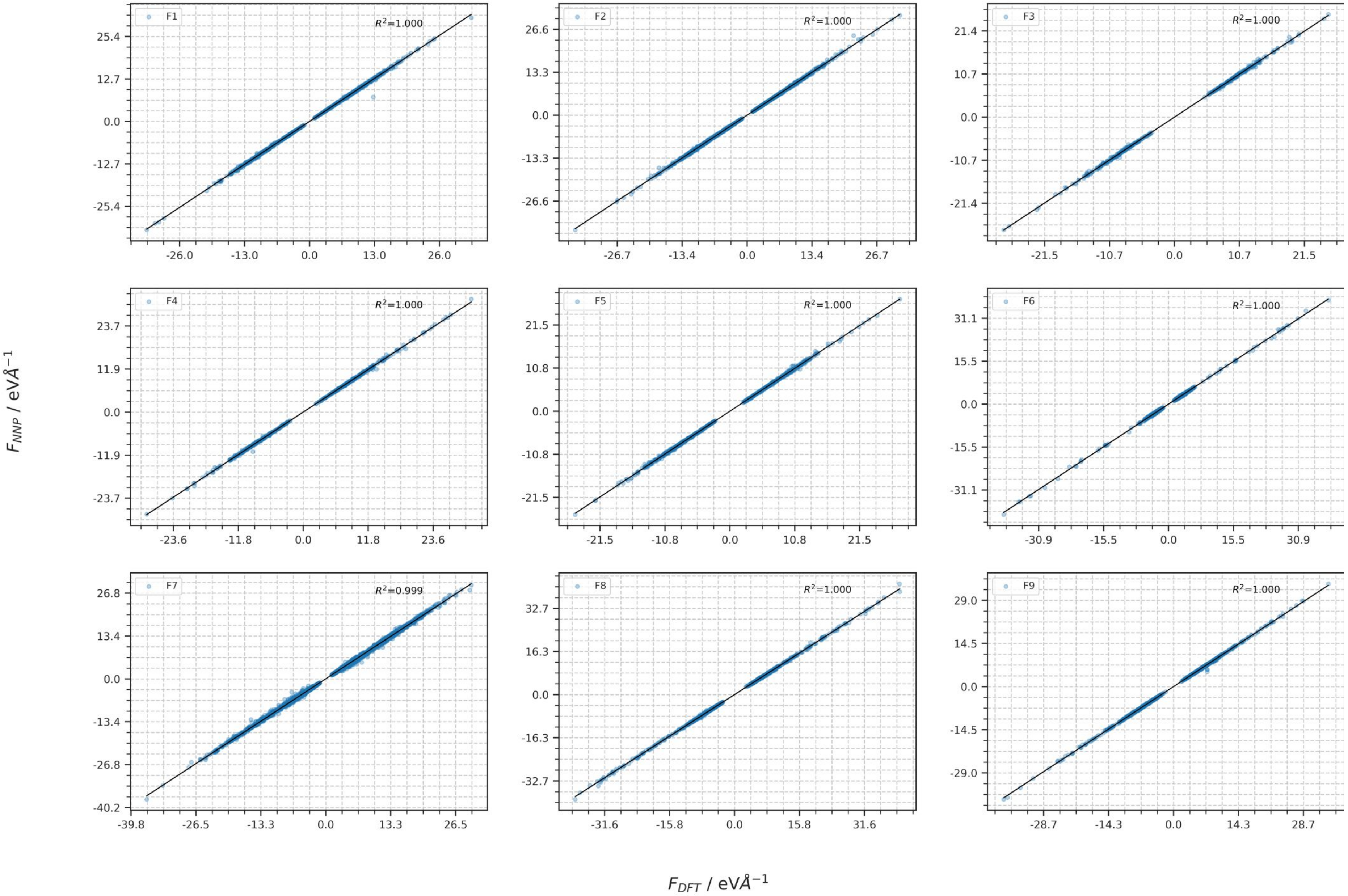

Figure S5 Correlation of between the DFT forces and the predictions of the NNP model force based on test set.

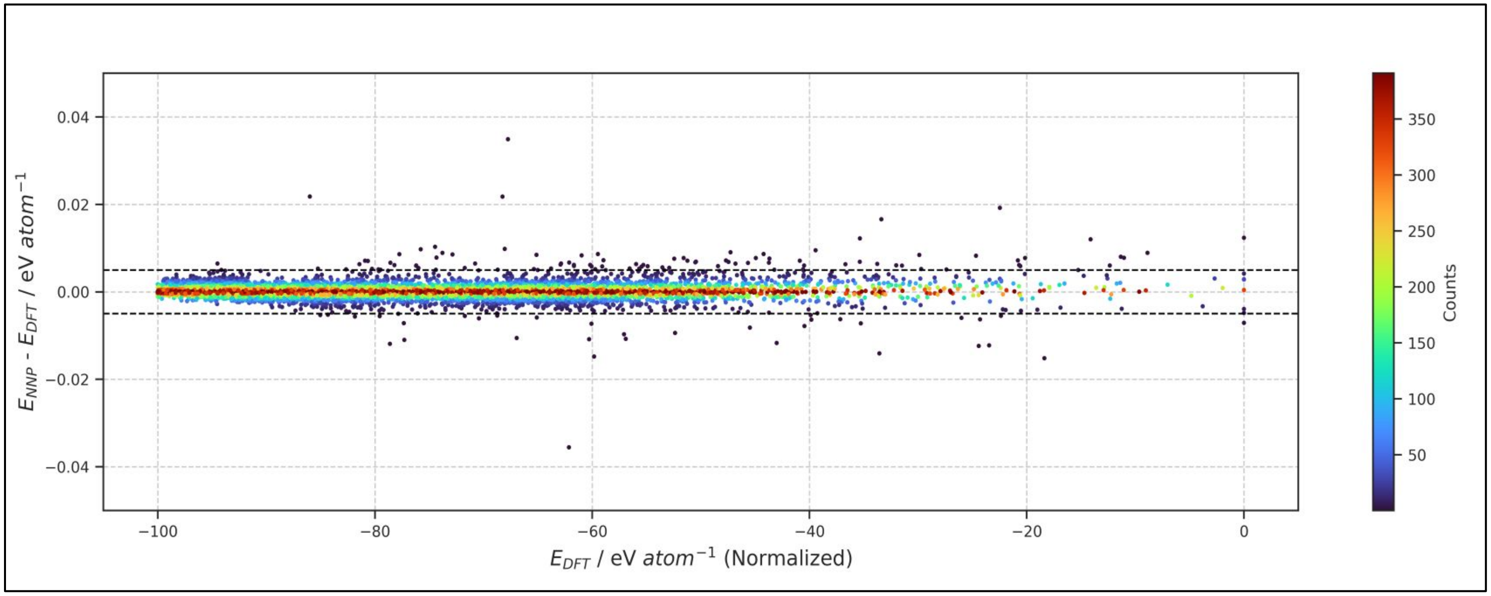

Figure S6 Errors in energies of fragments for test sets. The color bar shows the number of structures with the corresponding errors. Black dashed lines mark errors of 0.005 eV/atom. DFT energies (x axis) are normalized for a clear representation.

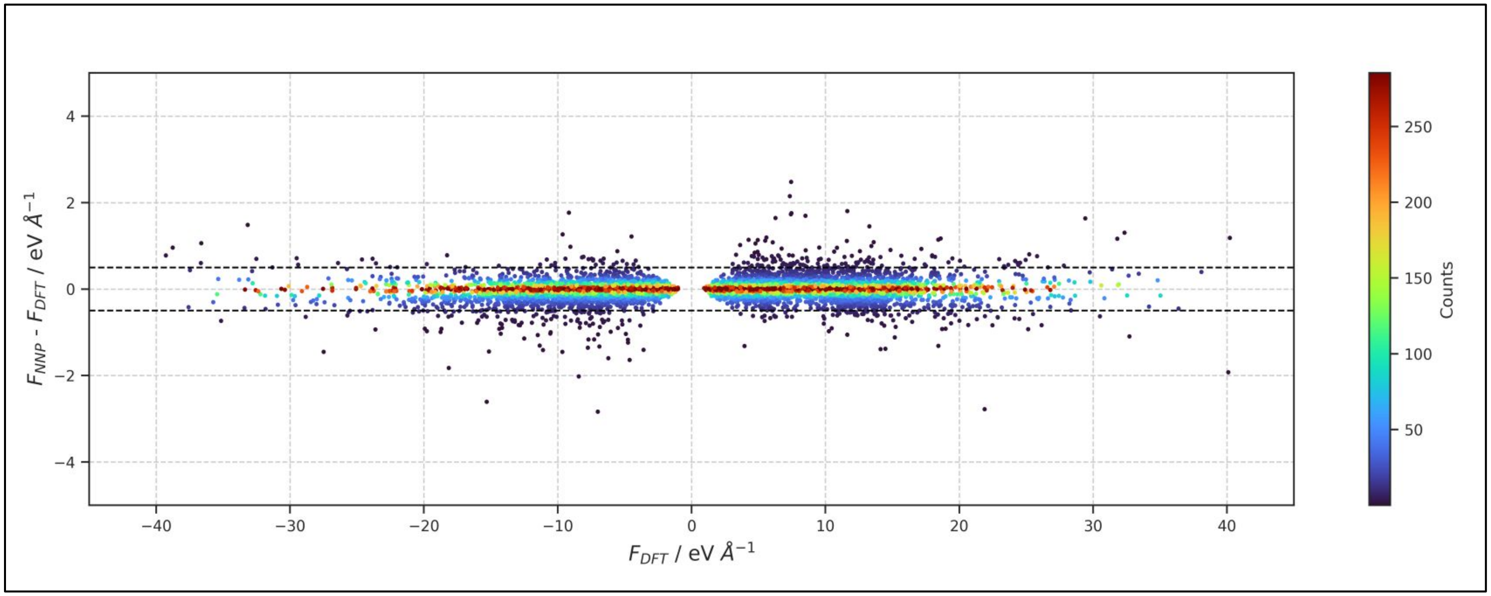

Figure S7 Errors in forces of fragments for test sets. The color bar shows the number of structures with the corresponding errors. Black dashed lines mark errors of 0.5 eV/ Å.

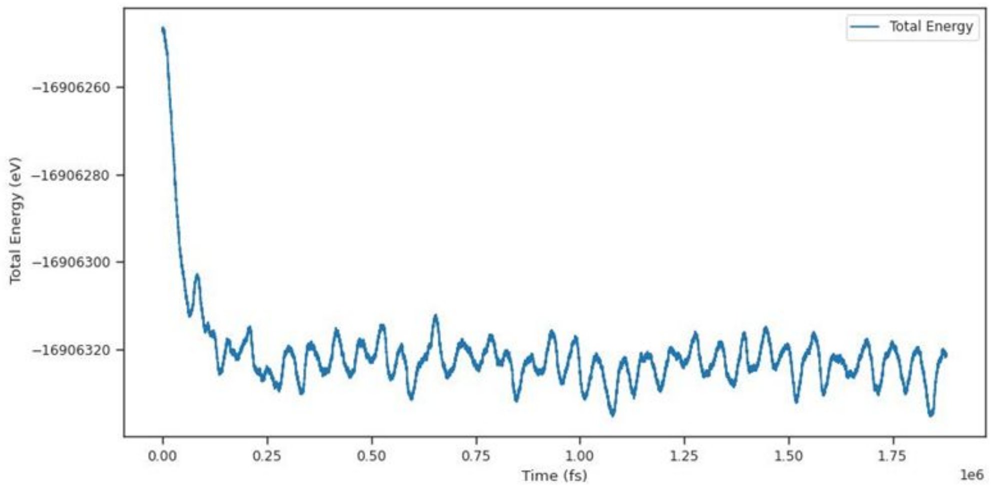

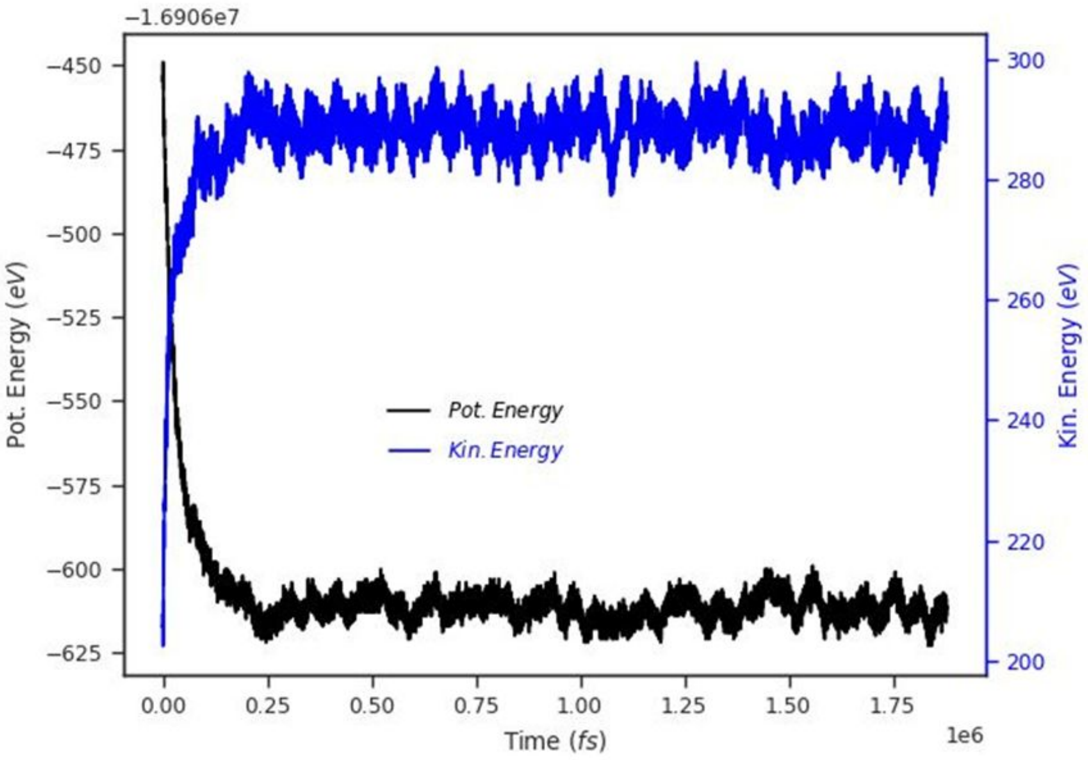

Figure S8: Potential, kinetic, and total energy terms conserved during NVT-MD simulation at 100 bar and 300K for IRMOF-1 with HDNNP.

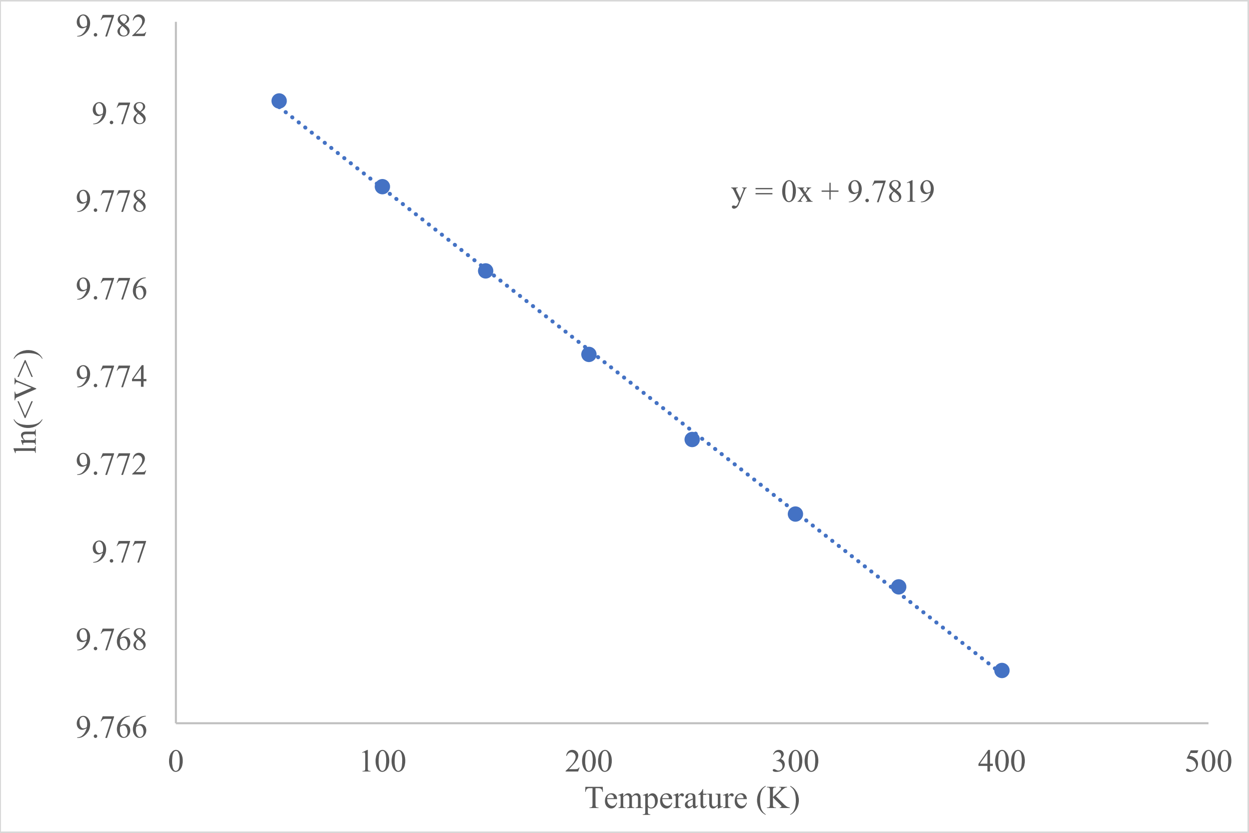

Figure S9: ln(<V>) vs. T (in Kelvin) plot of IRMOF-1. The slope, corresponding to negative thermal expansion (NTE) coefficient (α_0) is -36.9E-6 well agreement with experimental value of -39E-6 (as reported in Ref. 49 in manuscript)

a)

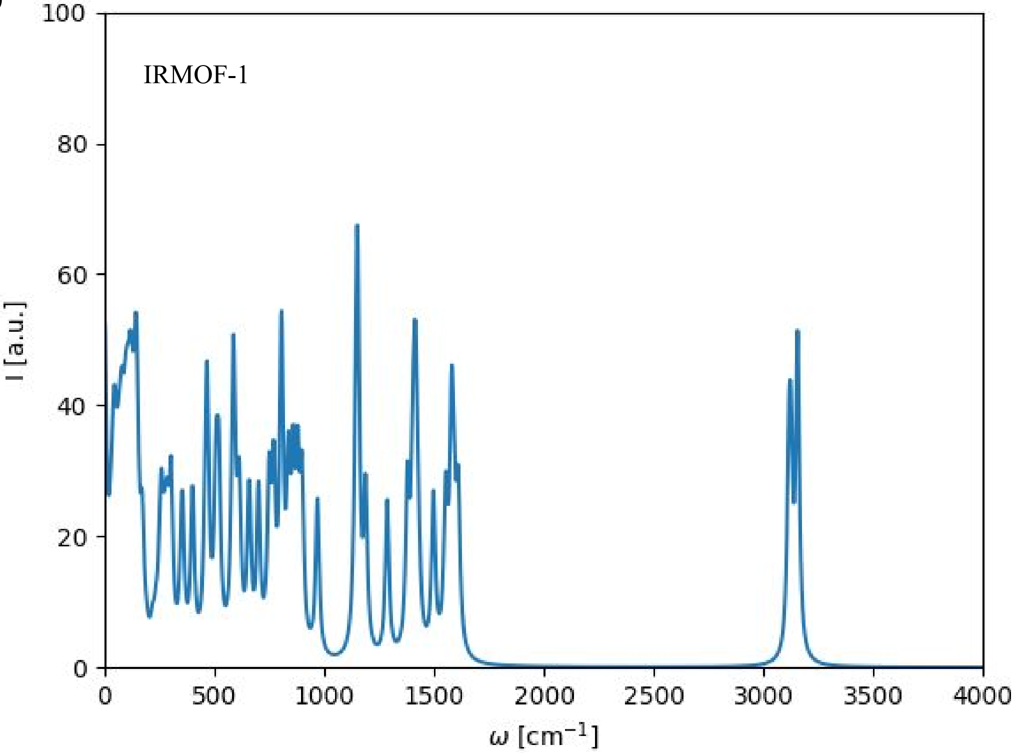

b)

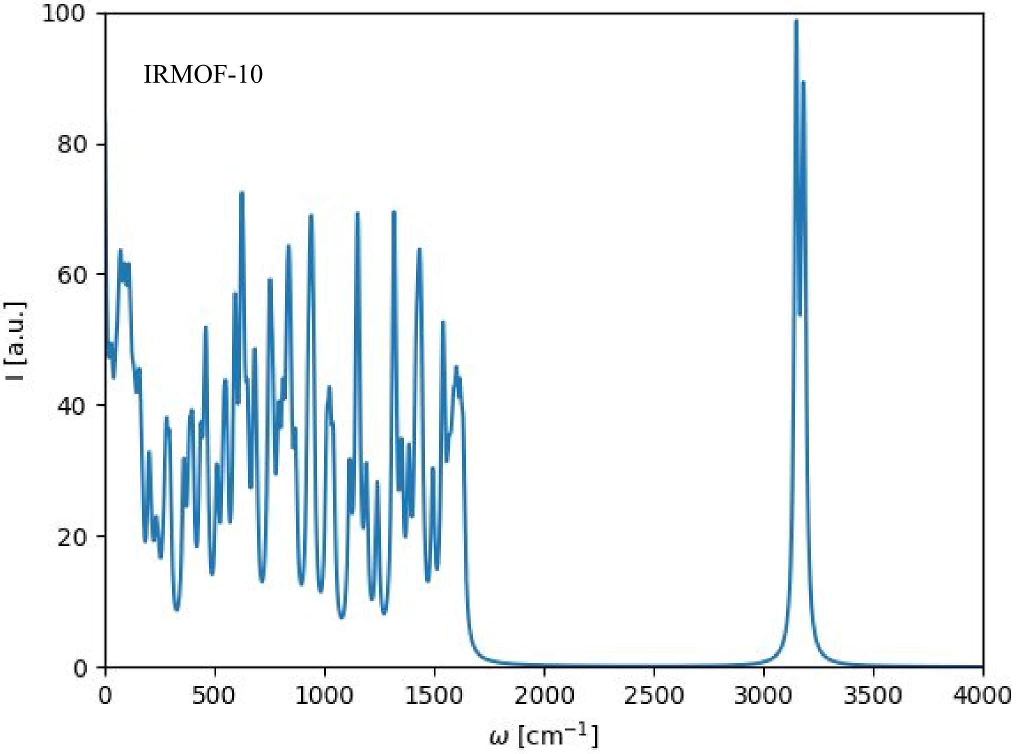

Figure S10: Phonons produced by calculation of vibrational modes using finite differences for a) IRMOF-1 and b) IRMOF-10 by HDNNP.

Table S1. LJ Potential Parameters for Gases and Frameworks.

	Force Field Type	ε (kcal/mol)	σ (Å)
Gases	H_2	0.067	2.96
	CH_4	0.294	3.73
Frameworks	Zn	0.124	2.46
	O	0.06	3.11
	C	0.106	3.43
	H	0.044	2.57

a)

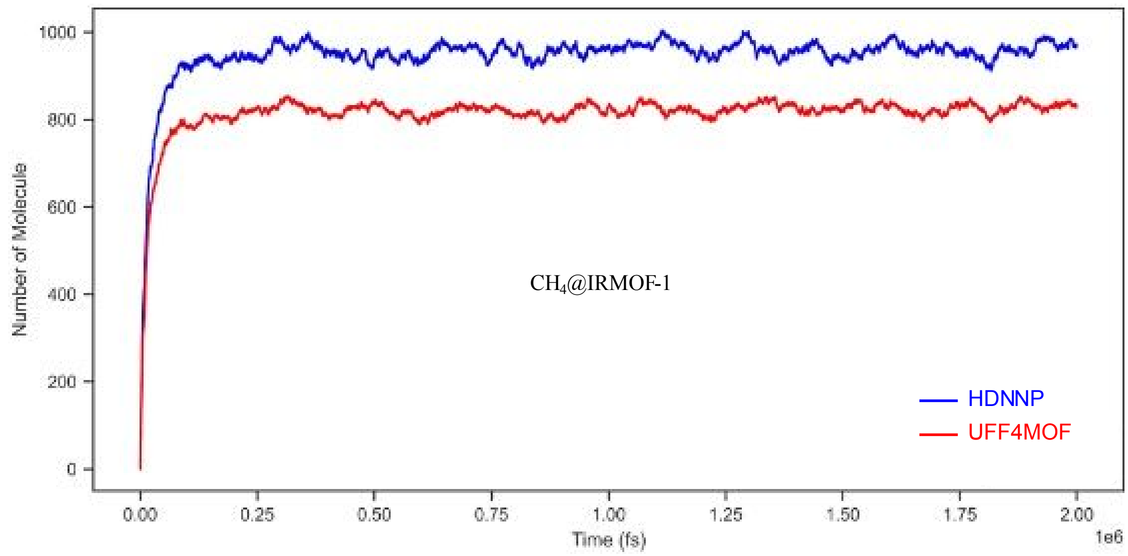

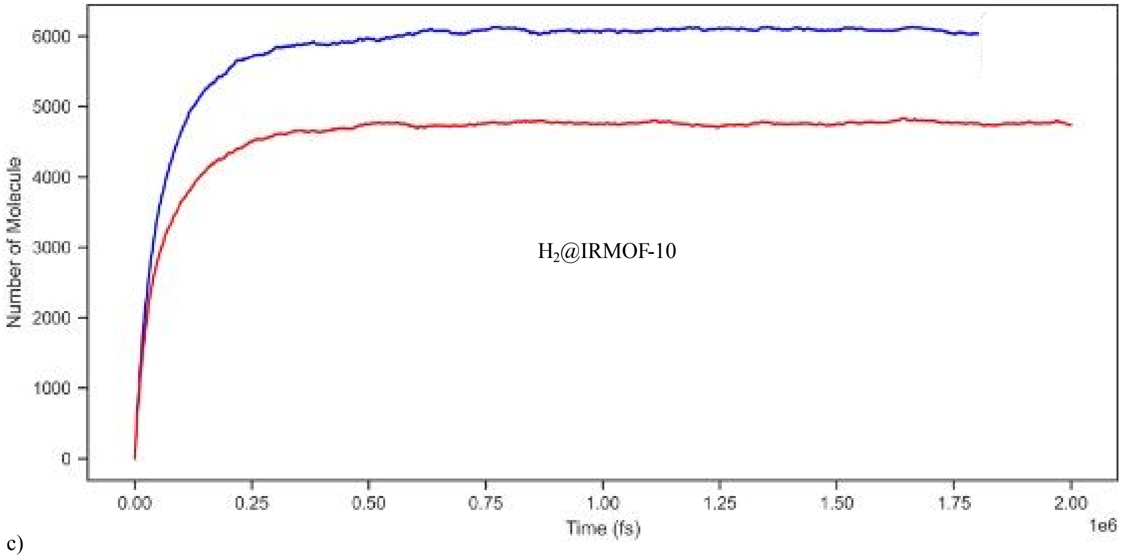

c)

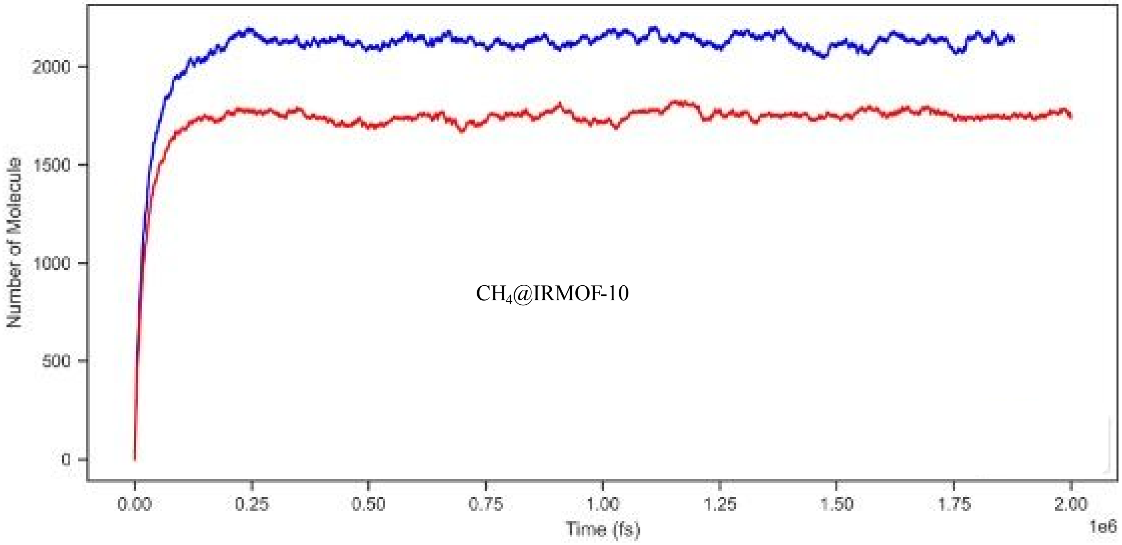

Figure S11 The number of gas loading during GCMC/MD simulation at 100 bar and 77 K for H_2, 300K for CH_4.

a)

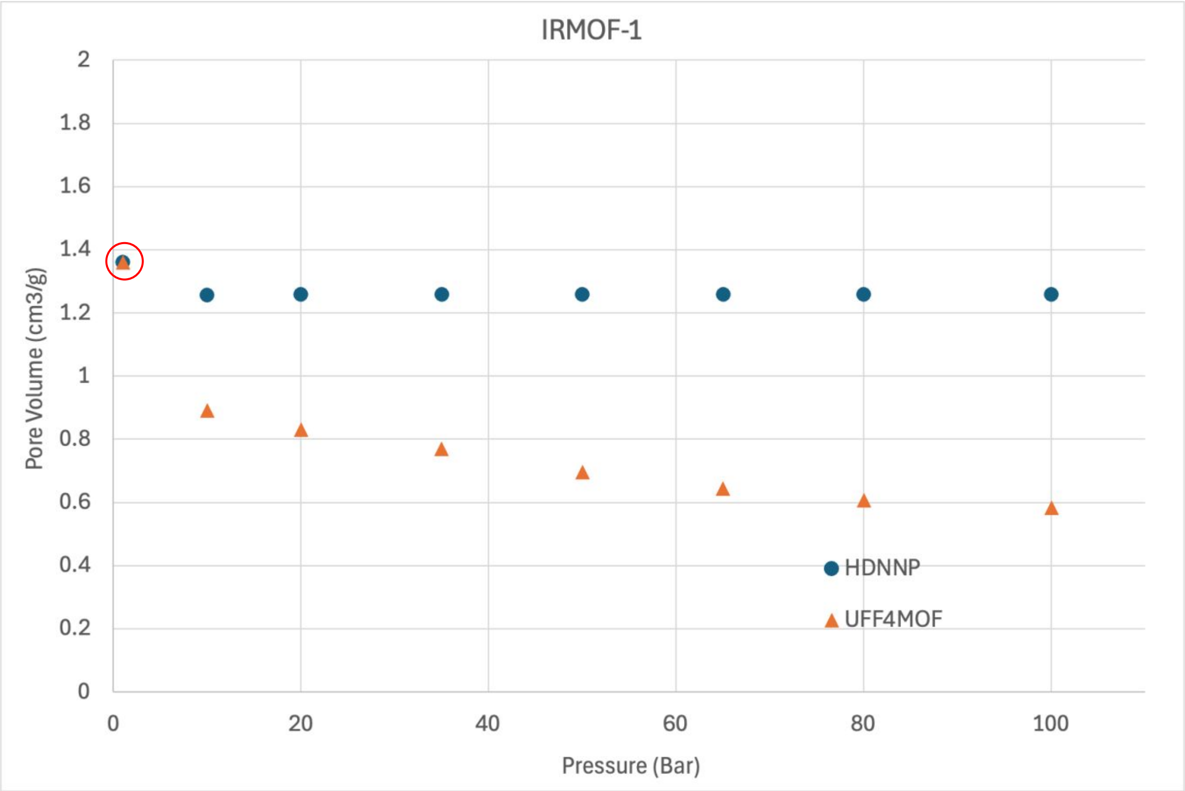

b)

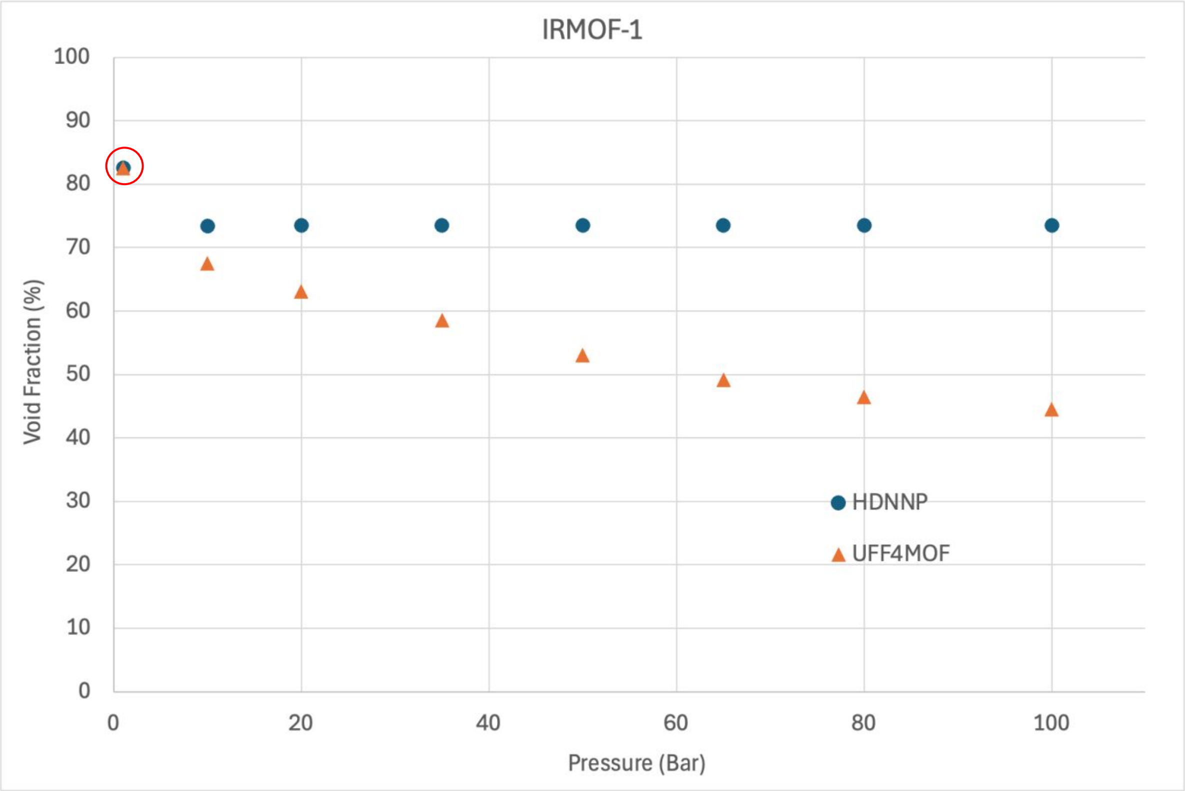

Figure S12: Mean values of a) pore volume and b) void fraction for CH_4@IRMOF-1 at 300K and different pressures from adsorption-relaxation MD simulations. Dashed circles represent initial structures.

a)

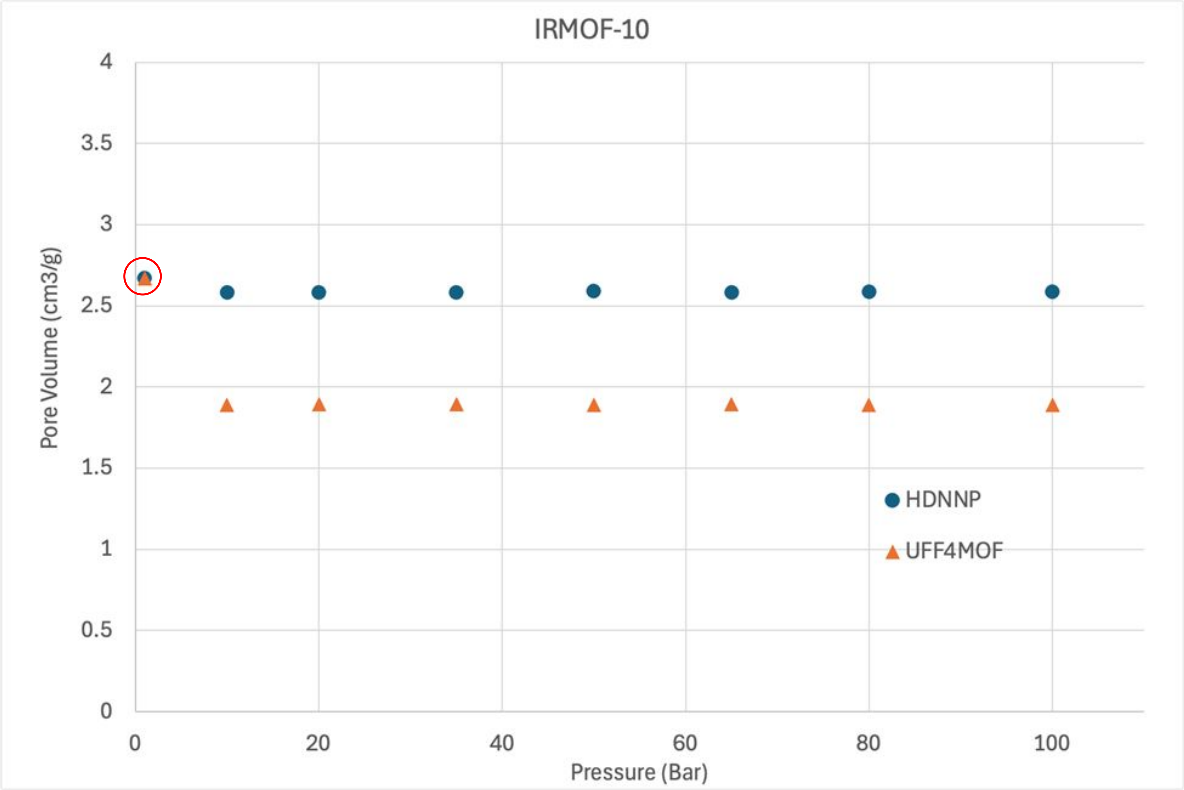

b)

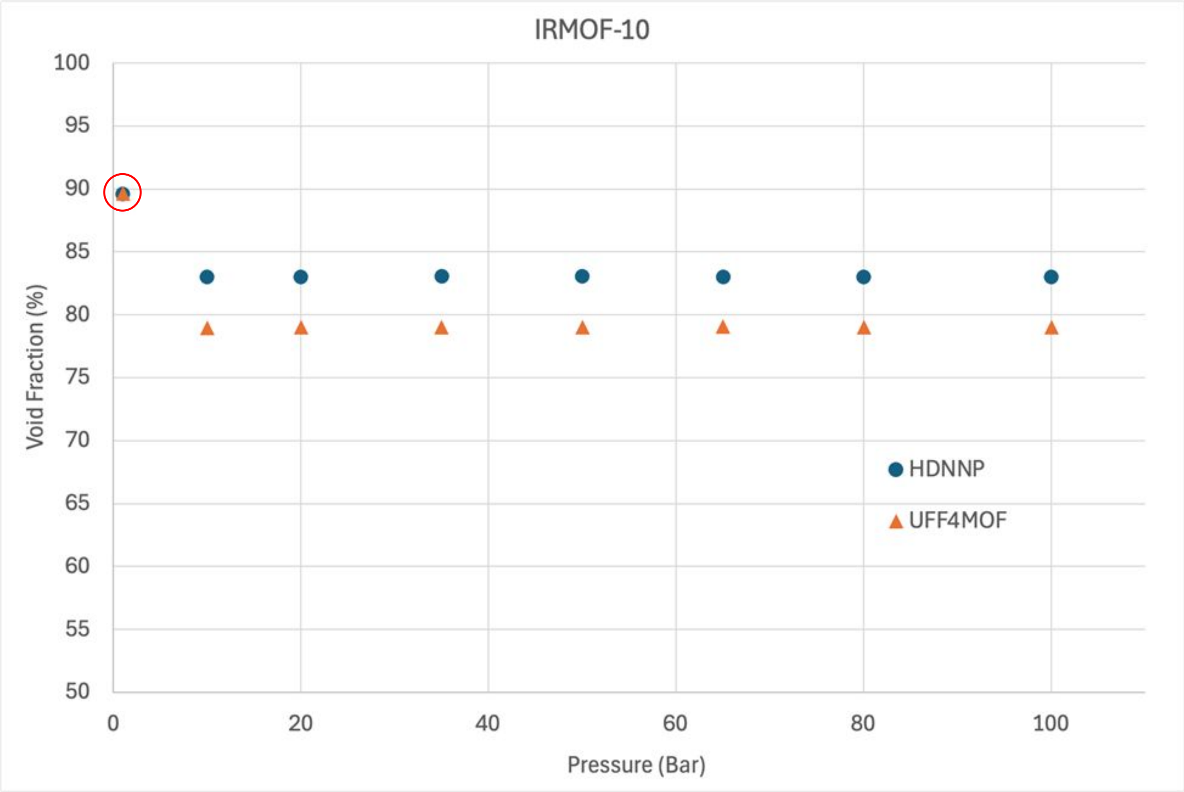

Figure S13: Mean values of a) pore volume and b) void fraction for CH_4@IRMOF-10 at 300K and different pressures from adsorption-relaxation MD simulations. Dashed circles represent initial structures.

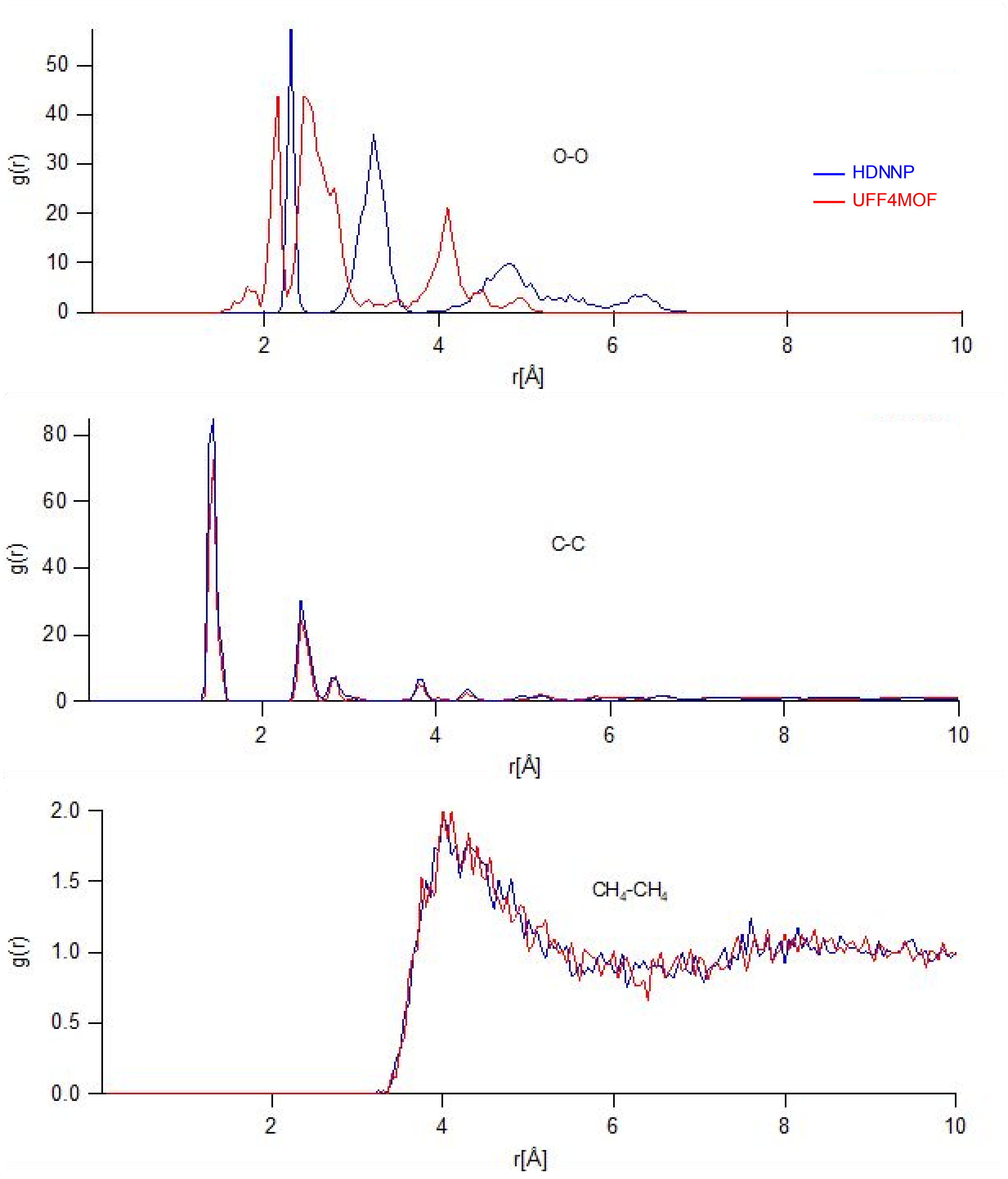

Figure S14 Radial distribution function for O-O and C-C atom pairs during adsorption relaxation simulations of CH_4 on IRMOF-10.
